# Supplementary material for: Condom use and HIV testing among adults in Switzerland: repeated national cross-sectional surveys 2007, 2012, and 2017
Source: BMC Public Health. 2023 Nov 3;23:2158. doi: 10.1186/s12889-023-17056-x (PMC10625239; doi:10.1186/s12889-023-17056-x)
Supplement: Supplementary file 1 — Supplementary Material 1 [file 12889_2023_17056_MOESM1_ESM.pdf]

Additional file 1: Supplementary tables S1-S16

Condom use and HIV testing among adults in Switzerland: repeated national cross-sectional surveys 2007, 2012, and 2017

Diana Buitrago-Garcia[1,2], Georgia Salanti[1], Nicola Low[1*]

1. Institute of Social and Preventive Medicine, University of Bern, Bern, Switzerland
2. Graduate School of Health Sciences, University of Bern, Bern, Switzerland

Table of Contents

Table S1.Filtering questions to determine eligible respondents to questions about condom use at last intercourse and HIV testing, according to survey year. 3

Table S2. Type of partner in men and women reporting any same-sex partner 2007,2012,2017 3

Table S3. Condom use at last sexual intercourse, men and women with only opposite-sex partners. Denominators, unweighted and weighted, for each survey and variable 4

Table S4. Condom use, men and women reporting only opposite-sex partners, 2007. Prevalence and associations with sociodemographic factors, sexual behaviours and substance use 7

Table S5. Condom use, men and women reporting only opposite-sex partners, 2012,2017. Prevalence and associations with sociodemographic factors, sexual behaviours, and substance use 9

Table S6. Condom use at last sexual intercourse, men and women with any same-sex partners. Denominators, unweighted and weighted, and number of missing values for each survey and variable 11

Table S7. Prevalence of condom use, men reporting any same-sex partner 2007, 2012, 2017 and associations with sociodemographic factors, sexual behaviours and substance use from univariable logistic regression, and multivariable logistic regression, 2007 and, 2012, 2017 13

Table S8. Condom use in men reporting any same-sex partner 2012, 201.Multivariable logistic regression 2017 including marital status. 15

Table S9. Condom use, women reporting any same-sex partner. Associations with sociodemographic factors, sexual behaviours and substance use from univariable logistic regression, 2007, 2012, 2017 and from multivariable logistic regression 2007 and multivariable logistic regression 2012, 2017. 16

Table S10. Lifetime HIV testing, men and women with only opposite-sex partners. Denominators, unweighted and weighted, for each survey and variable 18

Table S11. Lifetime HIV testing and associations from univariable and multivariable logistic regression, by sociodemographic and behavioural characteristics, in men reporting only opposite-sex partners 2007, 2012 and 2017 20

Table S12. Prevalence of lifetime HIV testing and associations from univariable and multivariable logistic regression, by sociodemographic and behavioural characteristics, in women reporting only opposite-sex partners 2007, 2012 and 2017 22

Table S13. HIV testing in the last 12 months, men and women reporting only opposite-sex partners and men reporting any same-sex partner 2012, 2017. 23

Table S14. Lifetime HIV testing, men and women with any same-sex partners. Denominators, unweighted and weighted, and number of missing values for each survey and variable 24

Table S15. Lifetime HIV testing and associations from univariable and multivariable logistic regression, testing, by sociodemographic and behavioural characteristics in men reporting any male sex partner, 2007, 2012, 2017 26

Table S16. Lifetime HIV testing, women reporting any same-sex partner, 2007, 2012, 2017. Associations with sociodemographic factors, sexual behaviours and substance use from univariable logistic regression 27

Table S1.Filtering questions to determine eligible respondents to questions about condom use at last intercourse and HIV testing, according to survey year.

Year	Criteria to determine respondents to question "Did you use a male condom the last time you had sex?"[a]
2007	a. Age between 16 and 74 years<br>b. Reported ever having had sexual intercourse<br>c. Reported ever having used a male condom
2012	a. Age between 16 and 74 years.<br>b. Reported ever having had sexual intercourse<br>c. Reported ever having had sex during the last 12 months
2017	a. Age between 16 and 74 years<br>b. Reported ever having had sexual intercourse
	**Criteria to determine proportion of people who have had been tested for HIV in their lifetime, question "How many times have you been tested for AIDS?" "When was the last time?"**
2007	a. Age between 16 and 74 years.<br>b. Blood donors: apart from donating blood the number of HIV tests done or<br>c. Non-blood donors: the number of HIV tests done.<br>d. Year and month of first and last test.
2012	a. Participants older than 16 years<br>b. Blood donors: apart from donating blood the number of HIV tests done or<br>c. Non-blood donors: the number of HIV tests done.<br>d. Year and month the last test.
2017	a. Participants between 16 and 74 years.<br>b. Number of HIV tests done in life<br>c. Tests done within 12 months or more than 12 months ago.

Questions about sexual partners referred to the sexual lifetime for the surveys in 2007 and 2012 and the last five years for the 2017 survey.

Table S2. Type of partner in men and women reporting any same-sex partner 2007,2012,2017

**Sexual partners**	**2007**	**2012**	**2017**
	**n (%)**	**n (%)**	**n (%)**
**Men**			
**Total**	204 (32)	246(39)	183 (29)
Mainly with women, but at least one man	94 (36)	117 (46)	47 (18)
As many women as men	20 (37)	23 (43)	11 (20)
Mainly men but, at least one woman	43 (44)	43 (44)	12 (12)
Only with men	47 (21)	63 (28)	113 (51)
**Women**			
**Total**	227 (32)	300 (43)	172 (25)
Mainly with men, but at least one woman	173 (34)	250(48)	92 (18)
As many men as woman	22 (51)	17 (40)	4 (9)
Mainly women but, at least one man	25 (45)	23 (41)	8 (14)
Only with women	7 (8)	10 (12)	68 (80)

Table S3. Condom use at last sexual intercourse, men and women with only opposite-sex partners. Denominators, unweighted and weighted, for each survey and variable

	Men	Men	Men	Women	Women	Women
	**2007**	**2012**	**2017**	**2007**	**2012**	**2017**
**Totals, N unweighted, weighted[a]**	6416,229110	7382, 2427588	8049, 2724669	7688,2311227	7025, 2132003	8428, 2557958
**Age, years**						
16-24	554, 296442	966, 295120	903, 301043	528, 250661	851, 255515	876, 258899
25-34	984, 395552	1,064, 454261	1111, 528079	1210, 409123	1130, 419164	1291, 505920
35-44	1574, 527561	1,470, 488511	1448, 530176	1817, 535968	1571, 471335	1625, 509388
45-54	1232, 460546	1,705, 550479	1864, 573081	1381, 454705	1784, 504578	1960, 558324
55-64	1206, 373066	1,267, 390680	1520, 465839	1527, 397531	1086, 307860	1604, 434936
65-74	866, 245944	910, 248536	1203, 326452	1225, 263240	603, 173550	1072, 290492
**Region**						
Lake Geneva	61, 427987	1317, 440506	1424, 508007	43, 436777	1290, 407290	1550, 479185
Midland	52, 518003	1435, 542392	1579, 587768	38, 522838	1389, 472081	1739, 593088
Northwest	56, 307767	1026, 325278	849, 352834	16, 320667	979, 291493	904, 351691
Zurich	40, 413510	740, 415267	880, 488010	26, 391749	663, 344353	921, 443955
East	29, 338110	1117, 369950	1528, 390425	20, 303656	994, 300103	1487, 341979
Central	36, 207440	1218, 229857	1227, 275223	20, 235853	1187, 217857	1252, 241036
Ticino	15, 86293	529, 104338	562, 122401	9, 99688	523, 98827	575, 107024
**Highest educational level**						
No school/primary	627, 260072	842, 274694	951, 292971	1188, 348046	894, 275746	1035, 288781
Secondary	3531, 1251313	3599, 1181527	3783, 1264045	5096, 1535478	4315, 1283197	4772, 1400556
Tertiary	2257, 787519	2924, 966760	3301, 1163362	1401, 426510	1797, 567319	2600, 860448
Missing	1, 206	17, 4608	14, 4291	3, 1193	19, 5740	21, 8173
**Income, SFr**						
No income	150, 64499	25, 177565	346, 112507	889, 293752	918, 279067	978, 271987
<4500	2183, 806982	2344, 790486	2689, 918762	4434, 1363467	4031, 1221547	4953, 1466363
4500-6000	1569, 539763	1718, 570588	1897, 654101	895, 249927	730, 222686	1049, 356014
>6000	1832, 652958	2242, 725455	2524, 850147	418, 112380	461, 132703	735, 251417
Missing	682, 234908	827, 263494	593, 189153	1052, 291702	885, 276000	713, 212177
**Marital status**						
Single	1843, 722189	2281, 849328	2407, 995481	1840, 587443	1798, 585228	2255, 795789
Married	3684, 1354831	4391, 328466	4916, 1447463	4091, 1357416	4400, 1262541	5105, 1414870
Widowed	153, 32443	84,28715	71, 24841	671, 120218	132, 40937	206, 59874
Divorced	734, 188300	575, 219908	655, 256884	1083, 245648	693, 241865	862, 287426
Missing	2, 1347	3, 1171	0,0	3, 502	2, 1431	0,0

**Sex partners last 12m**						
1	5035, 1848213	6382, 2095192	6498, 2158653	5916, 1933962	6583, 1995736	6960, 2094370
2-4	686, 247747	731, 243845	666, 255717	421, 126990	397, 121682	374, 127432
5+	205, 77071	228, 77697	199, 79313	35, 10850	29, 8387	30, 11663
Missing	490, 126080	41, 10854	686, 230987	1316, 239425	16, 6198	1064, 324494
**Sex frequency last 12m**	Not asked			Not asked		
1-2 per year	..	271, 80731	351, 118565	..	325, 93051	376, 111423
1 per month	..	828, 261277	1028, 329464	..	864, 249163	1124, 338486
2-3 per month	..	1472, 473009	1701, 546318	..	1283, 375995	1718, 511495
1 per week	..	1991, 650719	1944, 665376	..	1975, 607513	2090, 629519
2-3 per week	..	2267, 765183	1962, 693239	..	2071, 652754	1680, 531014
4+ per week	..	398, 149006	320, 118262	..	257, 79908	210, 70530
Missing	..	155, 47662	743, 253443	..	250, 73619	1219, 365490
**Partner type last intercourse**	Not asked			Not asked		
Stable	..	6665, 2190882	7173, 2393191	..	6714, 2040553	7982, 2409173
Occasional	..	677, 226436	803, 302565	..	306, 90071	433, 145050
Sex worker	..	31, 7646	67, 26208	..	0, 0	1, 200
Missing	..	9, 2585	6, 2705	..	5, 1380	12, 3535
**HIV testing**						
Never tested	5452, 1971699	4345, 1383746	4580, 1482996	6431, 1956232	3831, 1128432	4344, 1228391
>12m ago	624, 210219	2337, 801418	2857, 1017787	840, 239967	2443, 767295	3432, 1096425
Within last 12m	293, 98775	487, 171075	524, 197190	371, 102371	568, 182702	537, 199462
Missing	47, 18417	213, 71350	88, 26695	46, 12657	183, 53574	115, 33680
**Alcohol**						
No use	527, 188977	582, 218019	818, 285192	1384, 405764	1132, 342310	1533, 470954
Daily	1365, 425641	1261, 375407	1175, 352970	753, 197280	549, 152674	521, 143233
Weekly	3397, 1270669	4171, 1362093	4626, 1583047	2954, 932680	3137, 958014	3963, 1228355
Monthly	1122, 411170	1367, 471796	1428, 502750	2595, 774999	2206, 678812	2410, 715234
Missing	5, 2653	1, 273	2,711	2, 504	1, 193	1, 182
**Tobacco**						
Non-smoker	4236, 1503618	4891, 1575101	5488, 1807651	5670, 1712757	5175, 1557199	6315, 1889971
Casual smoker	619, 224346	726, 244321	776, 287648	463, 147473	486, 147355	605, 196721
Daily smoker	1561, 571147	1764, 606873	1785, 629371	1553, 450552	1364, 427449	1507, 471044
Missing		7, 1293	0, 0	2, 444	0, 0	1, 222
**Cannabis**[b]						
No use	4313, 1548932	5302, 1693509	5549, 1, 770092	5876, 1795362	5828, 1740879	6547, 1912407
>12m ago	1361, 509256	1525, 548295	1836, 683783	1105, 356307	959, 314830	1532, 523148

Within last 12m	319, 126221	534, 178092	633, 258879	121, 38680	221, 71061	333, 118541
Missing	423, 114702	21, 7693	31,11915	586, 120879	17, 5233	13, 3863
**Other illicit drugs[b, c]**						
No use	5683, 2076343	6958, 2275033	7469, 2479592	1182, 2137731	6798, 2058121	8123, 2444996
>12m ago	317, 108801	392, 140247	522, 217782	100, 56689	217, 70348	289, 106705
Within last 12m	13, 4404	22, 8031	45, 21281	5, 754	7, 2877	12, 5399
Missing	403, 109563	10, 4277	13, 6013	571, 116053	3, 656	4, 858

a. Overall denominators for condom use and HIV testing differ because eligibility criteria for answering each question differed;

b. In 2007 questions about drugs were only asked to people between 15 to 69 years;

c. Combines use of ecstasy, cocaine, or heroin

Abbreviations: m, months; SFr, Swiss Francs; yrs, years; .., question not asked.

Table S4. Condom use, men and women reporting only opposite-sex partners, 2007. Prevalence and associations with sociodemographic factors, sexual behaviours and substance use

**Condom Use**	**Men 2007**			**Women 2007**		
	**Prevalence (95% CI)**	**Unadjusted OR (95% CI)**	**Adjusted OR (95% CI)[a]**	**Prevalence (95% CI)**	**Unadjusted OR (95% CI)**	**Adjusted OR (95% CI) [a]**
**Total**	26 (24-27)			20 (18-21)		
**Age, years**		p<0.001	p<0.001		p<0.001	p<0.001
Median (IQR)[b]	29 (21-41)	0.93 (0.92, 0.93)	0.95 (0.94, 0.96)	32 (22-42)	0.94 (0.93, 0.94)	0.95 (0.94,0.96)
16-24	64 (59-69)	-	-	43 (38-48)	-	-
25-34	30 (26-33)	-	-	24 (21-26)	-	-
35-44	19 (17-22)	-	-	16 (14-18)	-	-
45-54	12 (10-14)	-	-	10 (8-12)	-	-
55-64	7 (5-9)	-	-	5 (3-6)	-	-
65-74	6 (4-8)	-	-	3 (2-5)	-	-
**Region**		p=0.9	p=0.9		p=0.004	p=0.15
Lake Geneva	23 (20-26)	reference	reference	15 (13-17)	reference	reference
Midland	22 (19-25)	0.94 (0.74, 1.19)	0.99 (0.74, 1.34)	15 (13-17)	1.04 (0.82, 1.32)	1.32 (0.97, 1.80)
Northwest	21 (18-25)	0.93 (0.70, 1.23)	1 (0.71, 1.41)	13 (10-15)	0.84 (0.62, 1.12)	1 (0.68, 1.47)
Zurich	22 (19-26)	0.97 (0.76, 1.25)	1.14 (0.82, 1.58)	19 (16-22)	1.34 (1.03, 1.73)	1.51 (1.07, 2.13)
East	23 (19-27)	1 (0.75, 1.34)	0.91 (0.63, 1.31)	16 (13-20)	1.13 (0.84, 1.54)	1.44 (0.98, 2.12)
Central	21 (17-25)	0.92 (0.69, 1.23)	0.94 (0.65, 1.35)	18 (15-21)	1.27 (0.97, 1.67)	1.31 (0.92, 1.88)
Ticino	4 (4-5)	1 (0.73, 1.36)	1.08 (0.71, 1.66)	4 (4-5)	0.74 (0.53, 1.03)	1.04 (0.66, 1.64)
**Highest education level**		p<0.001	p=0.3		p==0.001	p=0.086
No school/primary	39 (34-44)	reference	reference		reference	reference
Secondary	22 (20-24)	0.43 (0.34, 0.55)	0.76 (0.53, 1.07)		0.78 (0.61, 1.00)	0.78 (0.61, 1.00)
Tertiary	17 (15-19)	0.32 (0.25, 0.41)	0.8 (0.54, 1.18)		1.08 (0.83, 1.43)	1.08 (0.83, 1.43)
**Income, SFr**		p<0.001	p=0.3		p=0.3	p=0.9
No Income	52 (42-62)	2.46 (1.62, 3.73)	1.1 (0.67, 1.81)	14 (11-17)	0.82 (0.63, 1.08)	1 (0.73, 1.37)
<4500	30 (28-33)	reference	reference	16 (15-18)	reference	reference
4500-6000	18 (16-20)	0.49 (0.40, 0.60)	0.8 (0.62, 1.03)	18 (15-21)	1.12 (0.90, 1.41)	0.96 (0.71, 1.29)
>6000	13 (12-15)	0.35 (0.28, 0.43)	0.81 (0.60, 1.09)	16 (12-20)	0.97 (0.70, 1.36)	1.12 (0.73, 1.71)
**Marital status**		p<0.001	p<0.001		p<0.001	p<0.001
Single	46 (43-49)	7.74 (6.44, 9.31)	2.14 (1.62, 2.82)	36 (33-39)	6.77 (5.61, 8.17)	6.77 (5.61, 8.17)
Married	10 (9-11)	reference	reference	8 (7-09)	reference	reference
Widowed	4 (2-7)	0.37 (0.18, 0.76)	0.18 (0.05, 0.68)	8 (5-12)	1.07 (0.68, 1.67)	1.07 (0.68, 1.67)
Divorced	18 (14-22)	1.93 (1.44, 2.58)	1.41 (0.97, 2.06)	15 (13-18)	2.21 (1.70, 2.88)	2.21 (1.70, 2.88)
**Age first intercourse, yrs**		p<0.001	p<0.001		p<0.001	p<0.001
Median (IQR)[b]	18 (16-19)	0.94 (0.91, 0.96)	1.08 (1.04, 1.11)	18 (16.0, 20.0)	0.94 (0.91, 0.97)	1.09 (1.05, 1.13)
<16	28 (25-32)	-	-	22 (19-25)	-	-
16-18	22 (20-25)	-	-	17 (15-19)	-	-
19-25	19 (17-21)	-	-	12 (11-14)	-	-
26-30	13 (08-19)	-	-	13 (08-20)	-	-
>30	16 (05-34)	-	-	15 (06-30)	-	-
**Sex partners last 12m**		p<0.001	p<0.001		p<0.001	p<0.001
1	15 (14-17)	reference	reference	12 (11-13)	reference	reference
2-4	53 (48-58)	6.41 (5.10, 8.04)	2.97 (2.22, 3.96)	49 (43-55)	6.77 (5.20, 8.81)	2.81 (2.06, 3.82)
5+	63 (54-71)	9.58 (6.55, 14.0)	3.43 (2.16, 5.44)	40 (20-63)	4.73 (1.97, 11.4)	3.04 (1.20, 7.67)
**HIV testing**		p=0.2	p=05		p<0.001	p=0.4
Never	22 (20-23)	reference	reference	15 (14-16)	reference	reference
>12m ago	24 (20-29)	1.18 (0.92, 1.50)	1.16 (0.85, 1.58)	19 (16-22)	1.32 (1.05, 1.66)	1.18 (0.89, 1.58)
Within last 12m	26 (20-33)	1.29 (0.91, 1.81)	0.86 (0.55, 1.32)	26 (21-32)	2.03 (1.51, 2.75)	1.15 (0.78, 1.68)
**Alcohol**		p<0.001	p=0.3		p<0.001	p=0.5
No use	25 (20-31)	reference	reference	13 (11-15)	reference	reference
Daily	10 (9-13)	0.34 (0.24, 0.50)	0.66 (0.43, 1.02)	7 (05-09)	0.48 (0.31, 0.74)	1.03 (0.58, 1.81)
Weekly	25 (23-27)	0.96 (0.70, 1.33)	0.82 (0.56, 1.20)	17 (15-19)	1.41 (1.10, 1.80)	1.12 (0.81, 1.57)

Monthly	24 (21-28)	0.94 (0.66, 1.34)	0.85 (0.56, 1.28)	18 (16-20)	1.46 (1.13, 1.87)	1.27 (0.91, 1.77)
**Tobacco**		p=0.023	p=0.2		p<0.001	p=0.02
Non-smoker	21 (19-22)	reference	reference	15 (14-16)	reference	reference
Casual smoker	25 (20-29)	1.26 (0.96, 1.64)	0.91 (0.66, 1.27)	24 (20-29)	1.86 (1.41, 2.45)	0.95 (0.65, 1.40)
Daily smoker	25 (22-27)	1.26 (1.05, 1.50)	0.78 (0.61, 1.00)	16 (14-18)	1.10(0.91, 1.34)	0.68 (0.51, 0.90)
**Cannabis**[c]		p<0.001	p=0.9		p<0.001	p=0.6
No use	19 (17-21)	reference	reference	15 (13-16)	reference	reference
>12m ago	29 (26-32)	1.51 (1.25, 1.81)	1.01 (0.79, 1.29)	23 (20-26)	1.01 (0.76, 1.34)	
Within last 12m	44 (38-51)	2.83 (2.09, 3.82)	1.12 (0.74, 1.71)	38 (28-48)	1.34 (0.78, 2.31)	
**Other illicit drugs**[c,d]		p=0.059	p=0.8		p<0.001	p=0.009
No use	22 (21-24)	reference	reference	16 (15-17)	reference	reference
>12m ago	32 (26-39)	1.78 (1.49, 2.14)	0.87 (0.57, 1.32)	33 (25-42)	1.76 (1.44, 2.15)	1.01 (0.76, 1.34)
Within last 12m	38 (8-77)	3.45 (2.56, 4.65)	1.12 (0.15, 8.12)	49 (00-100)	3.54 (2.27, 5.51)	1.34 (0.78, 2.31)

a. Multivariable model includes all variables in the table, p value from Wald test;

b. Median age of the proportion who used a condom;

c. In 2007 questions about drugs were only asked to people between 15 to 69 years;

d. Combines use of ecstasy, cocaine, or heroin.

Abbreviations: CI, confidence interval; m, months; OR, odds ratio; SFr, Swiss Francs; yrs, years.

Table S5. Condom use, men and women reporting only opposite-sex partners, 2012,2017. Prevalence and associations with sociodemographic factors, sexual behaviours, and substance use

	Men 2012		Men 2017		Women 2012	Women 2012	Women 2017	
**Condom use**	**Prevalence (95% CI)**	**Unadjusted OR (95% CI)**	**Prevalence (95% CI)**	**Unadjusted OR (95% CI)**	**Prevalence (95% CI)**	**Unadjusted OR (95% CI)**	**Prevalence (95% CI)**	**Unadjusted OR (95% CI)**
**Age, yrs**		p<0.001		p<0.001		p<0.001		p<0.001
Median (IQR)[a]	24 (22, 25)	0.92 (0.92, 0.93)	32 (24, 44)	0.94 (0.93, 0.94)	31 (22, 41)	0.93 (0.92, 0.93)	34 (25, 45)	0.94 (0.94, 0.95)
16-24	67 (63 -70)		64 (60 - 68)		47 (43-51)		48 (44-52)	
25-34	35 (31-39)		36 (36 - 43)		26 (22-9)		31 (28-34)	
35-44	20 (18-23)		27 (24 - 29)		18 (16 -20)		23 (21-25)	
45-54	13 (11- 15)		19 (17 - 21)		10 (9 -12)		15 (14-17)	
55-64	8 (6- 10)		11 (9 - 13)		3 (2 -5)		9 (7-11)	
65-74	5 (4- 8)		6 (4 - 7)		2 (1- 3)		4 (3-6)	
**Region**		p=0.4		p=0.8		p=0.13		p=0.009
Lake Geneva	25 (22-27)	reference	27 (25-30)	reference	15 (12-17)	reference	22 (20-25)	reference
Midland	22 (20-25)	0.88 (0.71, 1.10)	25 (23-28)	0.89 (0.74, 1.08)	18 (16-21)	1.33 (1.04, 1.71)	18 (16-20)	0.75 (0.62, 0.91)
Northwest	23 (20-27)	0.94 (0.74, 1.18)	26 (23-30)	0.94 (0.75, 1.17)	17 (15-21)	1.25 (0.95, 1.64)	22 (19-25)	0.97 (0.78, 1.21)
Zurich	24 (20-27)	0.96 (0.75, 1.23)	27 (24-30)	0.98 (0.79, 1.21)	19 (16-22)	1.36 (1.03, 1.79)	22 (19-25)	0.97 (0.78, 1.20)
East	23 (19-27)	0.93 (0.71, 1.21)	25 (23-28)	0.9 (0.74, 1.10)	18 (15-22)	1.29 (0.97, 1.72)	23 (20-25)	1 (0.82, 1.23)
Central	26 (23-30)	1.11 (0.88, 1.39)	28 (25-32)	1.03 (0.83, 1.28)	20 (17-23)	1.44 (1.12, 1.84)	22 (19-25)	0.96 (0.76, 1.21)
Ticino	20 (17-25)	0.79 (0.60, 1.05)	27 (23-31)	0.96 (0.75, 1.23)	18 (15-23)	1.33 (0.97, 1.82)	16 (13-20)	0.68 (0.52, 0.89)
**Highest education level**		p<0.001		p<0.001		p=0.025		p<0.001
No school/primary	36 (32-40)	reference	37 (34-41)	reference	20 (17-24)	reference	23 (21-27)	reference
Secondary	25 (23 - 27)	0.59 (0.48, 0.72)	28 (26-29)	0.64 (0.54, 0.77)	16 (15-18)	0.79 (0.62, 1.01)	19(18-20)	0.76 (0.63, 0.92)
Tertiary	18 (17- 20)	0.4 (0.33, 0.50)	23 (21-25)	0.64 (0.54, 0.77)	20 (17-22)	0.98 (0.74, 1.28)	23 (21-25)	0.99 (0.81, 1.21)
**Income, SFr**		p<0.001		p<0.001		p<0.001		p=0.3
No income	58 (49-65)	2.62 (1.85, 3.69)	58 (49- 65)	1.91 (1.46, 2.49)	21 (18- 24)	1.19 (0.95, 1.47)	24 (21- 28)	1.23 (1.02, 1.49)
<4500	34 (31-37)	reference	34 (31- 37)	reference	18 (17- 20)	reference	21 (19- 22)	reference
4500-6000	19 (16-21)	0.44 (0.36, 0.54)	19 (16- 21)	0.56 (0.47, 0.66)	17 (14- 21)	0.91 (0.69, 1.20)	22 (19- 25)	1.1 (0.91, 1.34)
>6000	15 (13 -17)	0.33 (0.28, 0.40)	15 (13- 17)	0.45 (0.38, 0.52)	18 (13- 23)	0.96 (0.69, 1.34)	20 (17- 25)	1.01 (0.80, 1.28)
**Marital status**		p<0.001		p<0.001		p<0.001		p<0.001
Single	46 (43- 48)	6.73 (5.72, 7.91)	48 (46- 51)	6.37 (5.54, 7.32)	36 (33-39)	4.92 (4.15, 5.84)	38 (35-40)	4.25 (3.69, 4.90)
Married	11 (10- 12)	reference	13 (12- 14)	reference	10 (9-11)	reference	12 (11-14)	reference
Widowed	11 (5-19)	0.97 (0.47, 2.03)	13 (6-26)	0.98 (0.38, 2.50)	10 (5-17)	0.96 (0.48, 1.95)	10 (6-16)	0.81 (0.47, 1.39)
Divorced	15 (11- 18)	1.5 (1.10, 2.05)	19 (16- 23)	1.6 (1.23, 2.07)	14 (11-17)	1.22 (0.87, 1.70)	19 (16-22)	1.68 (1.35, 2.10)
**Age first intercourse, yrs**		p<0.001		p=0.3		p<0.001		p=0.9
Per year increase		0.90 (0.88, 0.93)		0.99 (0.98, 1.01)		0.93 (0.90, 0.96)		1 (0.98, 1.02)
**Sex partners last 12m**		p<0.001		p<0.001		p<0.001		p<0.001
1	17 (16-18)	reference	19 (18-20)	reference	15 (14-16)	reference	18 (17-19)	reference
2-4	64 (59-69)	8.78 (7.03, 11.0)	61 (56-65)	6.55 (5.33, 8.05)	55 (49-61)	6.92 (5.32, 9.00)	55 (49-61)	5.71 (4.46, 7.33)
5+	71 (62-80)	12.3 (7.97, 19.1)	73 (65-80)	11.6 (7.84, 17.2)	61 (35-83)	8.71 (3.38, 22.4)	56 (33-77)	5.81 (2.45, 13.8)
**Sex frequency last 12 m**		p<0.001		p<0.001		p<0.001		p<0.001
1-2 per year	54 (47-61)	2.53 (1.76, 3.63)	46 (40-53)	1.78 (1.32, 2.39)	38 (31-46)	2.24 (1.53, 3.28)	37 (32-43)	1.96 (1.45, 2.66)
1 per month	32 (27-36)	reference	33 (29-36)	reference	22 (18-25)	reference	23 (20-26)	reference
2-3 per month	27 (24-30)	0.81 (0.62, 1.04)	29 (27-32)	0.85 (0.69, 1.04)	21 (18-23)	0.94 (0.72, 1.23)	20 (18-23)	0.83 (0.67, 1.04)
1 per week	18 (16-20)	0.47 (0.36, 0.61)	23 (21-26)	0.62 (0.51, 0.77)	16 (14-18)	0.66 (0.51, 0.86)	18 (16-20)	0.74 (0.59, 0.91)
2-3 per week	20 (18-22)	0.54 (0.42, 0.70)	18 (16-20)	0.45 (0.36, 0.55)	15 (13-17)	0.62 (0.48, 0.80)	17 (15-20)	0.7 (0.55, 0.87)
4+ per week	24 (18-29)	0.66 (0.45, 0.96)	21 (16-27)	0.56 (0.39, 0.81)	12 (7-20)	0.51 (0.27, 0.98)	17 (12-23)	0.68 (0.44, 1.04)
**Partner type last intercourse**		p<0.001		p<0.001		p<0.001		p<0.001
Stable	17 (16-19)	reference	20 (19- 21)	reference	15 (14-16)	reference	18 (17-19)	reference
Occasional	81 (77-85)	20.8 (15.8, 27.5)	75 (71-78)	12.2 (9.98, 14.9)	72 (65-78)	14 (10.1, 19.4)	68 (62-73)	9.46 (7.37, 12.1)
Sex worker	85 (66-95)	26.1 (9.52, 71.4)	87 (74-95)	26.3 (11.1, 62.8)	Not asked	-	Not asked	-
**HIV testing**		p<0.001		p<0.001		p<0.001		p<0.001
Never tested	24 (22-25)	reference	26 (24-28)	reference	18 (16-19)	reference	20 (18- 21)	reference
>12m ago	21 (18-23)	0.84 (0.71, 0.99)	25 (23-27)	0.95 (0.84, 1.09)	16 (14-18)	0.91 (0.77, 1.08)	20 (19-22)	1.03 (0.90, 1.17)
Within last 12m	37 (31-43)	1.9 (1.45, 2.50)	38 (33-43)	1.77 (1.41, 2.22)	25 (21-30)	1.58 (1.23, 2.04)	31 (27-36)	1.83 (1.45, 2.31)
**Alcohol**		p<0.001		p=0.001		p<0.001		p<0.001

No use	28 (23-34)	reference	31 (27-35)	reference	14 (12-17)	reference	23 (20-25)	reference
Daily	13 (10-15)	0.37 (0.26, 0.52)	11 (9-14)	0.28 (0.21,0.38)	6 (4-9)	0.42 (0.27, 0.65)	9 (7-13)	0.35 (0.24,0.52)
Weekly	25 (23-27)	0.86 (0.65, 1.14)	27 (26-29)	0.83 (0.68,1.01)	18 (16-20)	1.33 (1.04, 1.69)	20 (19-22)	0.87 (0.74, 1.04)
Monthly	26 (22-29)	0.88 (0.64, 1.20)	32 (29-35)	1.06 (0.84, 1.32)	22 (19-24)	1.67 (1.30, 2.15)	23 (21- 25)	1.02 (0.84, 1.22)
**Tobacco**		p=0.11		p=0.11		p<0.001		p<0.001
Non-smoker	22 (20-23)	reference	26 (24-27)	reference	17 (15-18)	reference	20 (19-21)	reference
Casual smoker	29 (24-34)	1.44 (1.12, 1.86)	30 (26-34)	1.24 (1.01, 1.51)	25 (21-30)	1.68 (1.29, 2.19)	31 (26- 35)	1.79 (1.45, 2.22)
Daily smoker	26 (23-29)	1.27 (1.07, 1.49)	26 (24-29)	1.02 (0.88, 1.19)	19 (17-22)	1.21 (0.99, 1.48)	21 (19-24)	1.11 (0.94, 1.31)
**Cannabis**		p<0.001		p<0.001		p<0.001		p<0.001
No use	24 (23-26)	reference	24 (23-26)	reference	16 (15-17)	reference	19 (18-21)	reference
>12m ago	27 (25-30)	1.17 (0.97, 1.41)	27 (25-30)	1.15 (0.99, 1.34)	21 (18-25)	1.42 (1.15, 1.75)	23 (21-26)	1.28 (1.09, 1.49)
Within last 12m	39 (34-43)	2.97 (2.35, 3.74)	39 (35-44)	1.99 (1.61, 2.44)	38 (30-47)	3.17 (2.17, 4.63)	37 (31-43)	2.41 (1.83, 3.16)
**Other illicit drugs**[b]		p<0.001		p<0.001		p=0.016		p=0.075
No use	23 (22-24)	reference	26 (25-27)	reference	18 (16-19)	reference	21 (20-22)	reference
>12m ago	30 (24-37)	1.48 (1.11, 1.97)	30 (26-35)	1.24 (0.98, 1.57)	26 (19-33)	1.62 (1.09, 2.41)	23 (18-29)	1.15 (0.82, 1.60)
Within last 12m	60 (29-87)	5.12 (1.66, 15.7)	36 (22-53)	1.63 (0.84, 3.17)	45 (0- 99)	3.89 (0.75, 20.3)	54 (9-94)	4.41 (1.17, 16.6)

d. Median age of the proportion who used a condom;

e. Combines use of ecstasy, cocaine, or heroin.

Abbreviations: CI, confidence interval; IQR, interquartile range; m, months; OR, odds ratio, CHF, Swiss Francs; yrs, years.

Table S6. Condom use at last sexual intercourse, men and women with any same-sex partners. Denominators, unweighted and weighted, and number of missing values for each survey and variable

Condom use	Men	Men	Men	Women	Women	Women
	2007	2012	2017	2007	2012	2017
**Totals, N unweighted, weighted[a]**	204,70271	245, 84238	183, 76438	220,66759	300, 96257	172, 69018
**Age, years**						
16-24	7, 3497	20, 5643	25, 8197	18, 8593	55, 15295	35, 14084
25-34	47, 19528	34, 14478	32, 17291	49, 17849	80, 31574	55, 25382
35-44	63, 21425	68, 27822	40, 18722	66, 15531	66, 20741	30, 12090
45-54	40, 12407	62, 19514	38, 16023	59, 18436	65, 18851	36, 12713
55-64	33, 10115	40, 11544	37, 13623	22, 5006	24, 6718	10, 3117
65-74	14, 3299	21, 5235	11, 2581	6, 1344	10, 3077	6, 1631
**Region**						
Lake Geneva	32, 11958	45, 15147	46, 19171	55, 14654	64, 19810	43, 16336
Midland	55, 18173	36, 15951	18, 8757	46, 11811	53, 18694	38, 18342
Northwest	18, 6636	44, 11119	20, 11332	33, 11938	59, 18221	16, 6521
Zurich	51, 20163	51, 24919	38, 19435	47, 18543	41, 21542	26, 14769
East	21, 7228	28, 9991	27, 10121	17, 5365	31, 8353	20, 6475
Central	14, 3722	34, 5939	26, 5903	18, 4010	37, 6536	20, 4442
Ticino	13, 2391	7, 1170	8, 1718	4, 437	15, 3100	9, 2133
**Highest educational level**						
No school/ primary	9, 3505	19, 4915	16, 5477	11, 3322	28, 8517	17, 5036
Secondary	108, 33417	111, 39221	83, 32537	131, 41629	160, 54460	83, 33636
Tertiary	87, 33349	115, 40102	84, 38423	78, 21808	112, 33279	72, 30346
Missing	0, 0	0, 0	0, 0	0, 0	0, 0	0, 0
**Income, SFr**						
No Income	5, 2205	9, 2488	12, 3984	15, 5635	20, 6896	8, 2581
<4500	76, 27183	70, 22070	64, 26740	122, 37141	184, 59501	106, 44156
4500-6000	53, 18533	49, 16453	45, 19462	43, 13094	39, 11864	31, 10373
>6000	52, 17628	85, 32340	50, 20141	23, 5677	29, 9141	22, 9965
Missing	18, 4723	32, 10885	12, 6110	17, 5212	28, 8854	5, 1943
**Marital status**						
Single	131, 44281	121, 46054	122, 52867	118, 33800	149, 49696	111, 47453
Married	44, 16380	101, 28417	18, 5042	46, 18141	105, 29705	22, 7883
Widowed	0, 0	1, 178	0, 0	4, 839	5, 1679	2, 428
Divorced	28, 8496	22, 9589	14, 5571	52, 13978	40, 14608	14, 5518
Missing	1, 1113	0, 0	29, 12956	0, 0	1, 569	23, 7735
**Age first intercourse, yrs**						
<16	59, 23916	94, 34200	70, 29812	89, 29114	162, 50085	76, 30797
16-18	67, 22018	80, 28715	64, 26995	77, 24899	87, 29698	53, 22093
19-25	67, 21340	64, 19584	44, 17456	48, 11575	44, 14199	40, 14872
26-30	4, 990	3, 746	3, 1175	3, 420	1, 183	2, 1003
>30	2, 393	0, 0	0, 0	2, 230	2, 830	0, 0
Missing	5, 1613	2, 992	1, 998	1, 521	2, 1261	1, 254
**Sex partners last 12m**						
1	82, 31018	163, 53952	89, 36364	135, 44408	241, 79105	106, 44848
2-4	67, 22612	48, 18743	41, 19755	37, 10859	45, 13384	41, 16743
5+	29, 10790	29, 10335	38, 15068	14, 3568	14, 3767	7, 3005
Missing	26, 5850	4, 1208	15, 5250	34, 7923	0, 0	18, 2506
**Sex frequency last 12m**	**Not asked**			Not asked		
1-2 per year	..	11, 3497	13, 3918	..	22, 6591	8, 3402
1 per month	..	27, 7807	26, 10429	..	32, 10029	29, 11156
2-3 per month	..	51, 16847	43, 19311	..	54, 19159	32, 12295
1 per week	..	58, 16937	41, 18896	..	69, 21662	38, 15715

2-3 per week	..	76, 33157	39, 16550	..	83, 27004	31, 15140
4+ per week	..	17, 4665	7, 2827	..	33, 10111	16, 6888
Missing	..	5, 1327	14, 4504	..	7, 1701	18, 4422
**Partner type, last intercourse**	**Not asked**			Not asked		
Stable	..	188, 67115	102, 43535	..	255, 84285	137, 54869
Occasional	..	53, 16300	76, 31278	..	45, 11972	33, 13418
Sex worker	..	4, 822	5, 1625	..	0, 0	0, 0
Missing	..	0, 0	0, 0	..	0, 0	2, 731
**HIV testing**						
Never tested	105, 36157	61, 16837	37, 12357	141, 42611	81, 27912	56,21757
>12m ago	51, 17619	111, 40139	80, 33188	48, 13631	158, 48215	80, 33248
Within last 12m	44, 15623	66, 24807	61, 28525	28, 9144	52, 16840	36, 14012
Missing	4, 871	7, 2454	5, 2367	3, 1373	9, 3289	0, 0
**Alcohol**						
No use	19, 4496	28, 8021	13, 4645	21, 7475	33, 10778	24, 10064
Daily	39, 14549	37, 12283	30, 10228	24, 6480	24, 7291	11, 4582
Weekly	104, 35320	137, 48188	101, 46129	116, 34066	161, 49932	93, 36322
Monthly	42, 15906	43, 15746	39, 15436	58, 18487	82, 28256	44, 18050
Missing	0, 0	0, 0	0, 0	1, 252	0, 0	0, 0
**Tobacco**						
Non-smoker	110, 40849	141, 48931	106, 45271	106, 30958	143, 49064	84, 34367
Casual smoker	26, 7455	31, 11905	18, 5952	22, 6517	51, 16581	19, 7859
Daily smoker	68, 21966	73, 23402	59, 25215	92, 29284	106, 30611	69, 26792
Missing	0, 0	0, 0	0, 0	0, 0	0, 0	0, 0
**Cannabis**[b]						
No use	97, 28824	129, 42310	94, 38959	89, 25715	103, 33352	74, 28862
>12m ago	66, 27309	74, 25507	56, 25133	94,28964	133, 44220	65, 26828
Within last 12m	31, 10391	40, 15510	31, 11225	34,10532	64, 18685	31, 12594
Missing	97, 28824	2, 910	2, 1121	3,1547	0	2, 734
**Other illicit drugs**[b, c]						
No use	149, 28824	185, 60328	148, 62088	169, 51011	234, 75008	136, 53648
Any use	46,17345	57, 23152	35, 14350	51, 15748	66, 21248	36, 15370
Missing	9, 3469	3, 757	0, 0	0, 0	0, 0	0, 0

a. Overall denominators for condom use and HIV testing differ because eligibility criteria for answering each question differed;

b. In 2007 questions about drugs were only asked to people between 15 to 69 years;

c. Combines use of ecstasy, cocaine, or heroin and combines categories of use more than 12 months ago and use within last 12 months.

Abbreviations: m, months; SFr, Swiss Francs; yrs, years; .., question not asked

Table S7. Prevalence of condom use, men reporting any same-sex partner 2007, 2012, 2017 and associations with sociodemographic factors, sexual behaviours and substance use from univariable logistic regression, and multivariable logistic regression, 2007 and, 2012, 2017

**Condom Use**	**2007**	**2007**	**2007**	**2012**	**2012**	**2017**	**2017**	
	**Prevalence (95% CI)**	**Unadjusted OR (95% CI)**	**Adjusted OR (95% CI)** [a]	**Prevalence (95% CI)**	**Unadjusted OR (95% CI)**	**Prevalence (95% CI)**	**Unadjusted OR (95% CI)**	**Adjusted OR (95% CI)**[a]
**Year**[a]								p=0.06
2007	40 (31-49)	-	-	-	-	-	-	..
2012	-	-	-	43 (34-53)	-	-	-	reference
2017	-	-	-	-	-	54 (46-63)	-	1.80 (0.97, 3.34)
**Age, years**		P=0.038	p=0.057		p<0.001		p<0.001	p<0.001
Median (IQR)[b]	36 (30-44)	0.97 (0.94-1.00)	0.97 (0.93, 1.00)	38 (29- 47)	0.95 (0.92, 0.97)	37 (26-48)	0.95 (0.93, 0.98)	0.96 (0.94, 0.98)
16-24	93 (00-100)	..	..	83 (52-98)	..	81 (57-95)	..	..
25-34	36 (22-53)	..	..	44 (25-64)	..	58 (37-78)	..	..
35-44	56 (39-71)	..	..	28 (16-42)	..	66 (49-81)	..	..
45-54	16 (08-30)	..	..	32 (19-47)	..	47 (28-66)	..	..
55-64	34 (15-59)	..	..	20 (7-38)	..	35 (18-56)	..	..
65-74	36 (11-72)	..	..	18 (3-46)	..	9 (0-49)	..	..
**Region**		p=0.9			p=0.4		p=0.9	
Lake Geneva	37 (19-58)	reference	..	31 (18-47)	reference	55 (39-70)	reference	..
Midland	44 (25-65)	1.36 (0.43, 4.36)	..	22 (09-42)	0.62 (0.20, 1.98)	54 (27-80)	0.99 (0.29, 3.32)	..
Northwest	33 (10-63)	0.84 (0.20, 3.55)	..	28 (13-47)	0.86 (0.30, 2.49)	58 (32-81)	1.12 (0.35, 3.55)	..
Zurich	41 (25-58)	1.17 (0.40, 3.44)	..	44 (29-59)	1.69 (0.68, 4.21)	51 (33-69)	0.86 (0.33, 2.21)	..
East	34 (12-63)	0.89 (0.22, 3.60)	..	41 (18-66)	1.5 (0.44, 5.04)	51 (27-75)	0.85 (0.27, 2.64)	..
Central	66 (25-94)	3.28 (0.56, 19.2)	..	23 (10-41)	0.65 (0.21, 1.95)	64 (41-83)	1.47 (0.49, 4.42)	..
Ticino	36 (6-77)	0.94 (0.16, 5.72)	..	48 (6-93)	2.06 (0.32, 13.4)	57 (15-92)	1.09 (0.21, 5.71)	..
**Highest education level**		p=0.09			p=0.2		p=0.5	
No school/primary	87 (68-105)	reference	..	47 (13-83)	reference	67 (13-83)	reference	..
Secondary	43 (30-55)	0.12 (0.02, 0.61)	..	27 (18-38)	0.41 (0.10, 1.71)	50 (18-37)	0.51 (0.14, 1.82)	..
Tertiary	34 (23-46)	0.08 (0.02, 0.42)	..	38 (28-50)	0.7 (0.17, 2.84)	56 (28-50)	0.64 (0.18, 2.29)	..
**Income, SFr**		p=0.075			p=0.2		P=>0.9	
No Income		17.6 (1.53, 203)	..	60 (0-100)	2.88 (0.53, 15.5)	46 (97-86)	0.72 (0.18, 2.93)	..
<4500		reference	..	34 (20-51)	reference	54 (40-69)	reference	..
4500-6000		0.69 (0.27, 1.76)	..	42 (27-60)	1.41 (0.54, 3.72)	57 (39-74)	1.1 (0.44, 2.76)	..
>6000		0.88 (0.36, 2.14)	..	27 (17-39)	0.71 (0.29, 1.73)	57 (41-72)	1.1 (0.48, 2.55)	..
**Marital Status**		p<0.001			p=<0.001		p=0.001	
Single	54 (42-65)	3.98 (1.53, 10.4)	..	38 (28-49)	1.88 (0.89, 3.98)	65 (54-74)	12.4 (3.00, 51.2)	..
Married	23 (10-40)	reference	..	25 (15-37)	reference	13 (3-42)	reference	..
Widowed	..	..	..	..	..	..	..	..
Divorced	14 (4-30)	0.54 (0.15, 1.96)	..	39 (15-69)	2.12 (0.59, 7.69)	44 (14-79)	5.30 (0.82, 34.1)	..
**Age first sexual intercourse, years**		p=0.6			p=0.2		p=0.4	
**Median (IQR)**	17 (15-19)	0.97 (0.88, 1.07)	..	17 (16-19)	0.93 (0.84, 1.05)	17 (16-18)	1.04 (0.94, 1.16)	..
**Sex partners 12m**		p=0.05	p=0.005		p<0.001		p<0.001	
1	22 (10-38)	reference	reference	18 (12-26)	reference	31 (20-44)	reference	Reference
2-4	53 (38-68)	4.09 (1.52, 11.0)	4.35 (1.64, 11.6)	47 (27-67)	3.86 (1.51, 9.87)	71 (52-86)	5.55 (2.02, 15.2)	1.94 (0.85, 4.42)
5+	64 (39-84)	6.3 (1.80, 22.1)	5.42 (1.50, 19.6)	84 (59-97)	22.6 (5.56, 91.9)	86 (72-95)	13.4 (4.62, 38.6)	5.83 (2.07,16.40)
**Average sex frequency 12 m**	Not asked				p=0.030		p=0.006	
1-2 times per year	..	..	..	75 (12-100)	3.10 (0.54, 18.0)	93 (68-100)	7.55 (1.08, 52.5)	..

Once per month	..	..	..	50 (26-74)	reference	65 (39-85)	reference	..
2-3 per month	..	..	..	32 (17-50)	0.47 (0.14, 1.55)	64 (46-79)	0.96 (0.29, 3.15)	..
Once per week	..	..	..	37 (22-53)	0.58 (0.19, 1.83)	42 (24-62)	0.4 (0.12, 1.38)	..
2-3t/week	..	..	..	25 (15-37)	0.33 (0.11, 1.00)	36 (20-55)	0.31 (0.09, 1.05)	..
4>/week	..	..	..	17 (3-42)	0.20 (0.04, 0.92)	77 (0-100)	1.78 (0.20, 15.8)	..
**Type of partner last sex intercourse**	Not asked				p<0.001		p<0.001	p<0.001
Stable partner	..	..	..	23 (16-31)	reference	33 (23-45)	reference	Reference
Occasional partner	..	..	..	74 (58-87)	9.85 (4.14, 23.5)	83 (73-90)	9.81 (4.46, 21.6)	5.98 (2.77, 12.90)
Sex-worker	..	..	..	88 (0-100)	25.1 (2.35, 270)	75 (0-100)	5.94 (0.61, 57.5)	8.29 (1.18, 58.30)
**HIV Testing**		p=0.051			p<0.001		p<0.001	
Never tested	31 (21-42)	reference	..	23 (10-39)	reference	57 (36-75)	reference	..
Tested >2m	44 (26-63)	1.79 (0.73, 4.37)	..	24 (15-34)	1.08 (0.41, 2.87)	43 (30-56)	0.57 (0.22, 1.47)	..
Tested in last 12m	61 (42-77)	3.48 (1.46, 8.30)	..	58 (42-73)	4.76 (1.70, 13.3)	63 (48-76)	1.46 (0.55, 3.90)	..
**Alcohol**		p=0.9			p=0.001		p=0.3	
No use	42 (15-72)	reference	..	61 (37-82)	reference	70 (33-94)	reference	..
Daily	35 (18-55)	0.76 (0.20, 2.83)	..	12 (4-27)	0.09 (0.02, 0.34)	40 (21-61)	0.29 (0.07, 1.28)	..
Weekly	43 (31-56)	1.07 (0.32, 3.65)	..	39 (28-49)	0.4 (0.15, 1.10)	55 (44-66)	0.53 (0.14, 2.03)	..
Monthly	40 (22-61)	0.95 (0.25, 3.65)	..	20 (9-37)	0.16 (0.05, 0.56)	58 (38-76)	0.6 (0.14, 2.61)	..
**Tobacco**		p= 0.031			p=0.6		p=0.2	
Non-smoker	33 (23-44)	reference	..	34 (24-44)	reference	55 (44-66)	reference	..
Casual smoker	58 (33-81)	2.87 (1.00, 8.27)	..	25 (9-47)	0.66 (0.22, 1.95)	74 (44-93)	2.26 (0.71, 7.19)	..
Daily smoker	50 (33-67)	2.02 (0.88, 4.64)	..	38 (25-51)	1.2 (0.59, 2.41)	48 (33-64)	0.76 (0.36, 1.63)	..
**Cannabis**[c]		p= 0.031			p=0.9		p=0.8	
No use	40 (28-53)	reference	..	32 (23-42)	reference	51 (40-63)	reference	..
>12m ago	32 (18-49)	0.71 (0.30, 1.69)	..	35 (22-49)	1.14 (0.55, 2.38)	56 (41-71)	1.22 (0.56-2.68)	..
Within last 12m	65 (43-84)	2.84 (1.04, 7.78)	..	32 (15-53)	1.01 (0.38, 2.64)	58 (37-78)	1.32 (0.51-3.39)	..
**Other illicit drugs**[b, c]		p= 0.089			p=0.2		p=0.5	
No use	37 (27-48)	reference	..	36 (28-45)	reference	53 (44-62)	reference	..
Any use	54 (36-71)	2.04 (0.89, 4.68)	..	26 (13-41)	0.61 (0.27, 1.37)	60 (41-77)	1.31 (0.58,3.10)	..

a. Prevalence of condom use for 2007 included. Multivariable model only includes 2012 and 2017 because denominator for 2007 excluded men who had never used a condom. Multivariable model includes age, number of sex partners and type of partner, p value from Wald test;

b. Median age of the proportion who used a condom;

c. In 2007 questions about drugs were only asked to people between 15 to 69 years;

d. Combines use of ecstasy, cocaine, or heroin and combines categories of use more than 12 months ago and use within last 12 months.

Abbreviations: CI, confidence interval; IQR, interquartile range; m, months; OR odds ratio; CHF, Swiss Francs; yrs, years; .., variable not included in multivariable model.

Table S8. Condom use in men reporting any same-sex partner 2012, 201.Multivariable logistic regression 2017 including marital status.

**Condom Use-MSM**	**2012**	**2017**	
	**Unadjusted OR (95% CI)**	**Unadjusted OR (95% CI)**	**Adjusted OR (95% CI)** [a]
**Year**			p=0.15
2012	-	-	reference
2017	-	-	1.64 (0.83, 3.24)
**Age, years**	p<0.001	p<0.001	p<0.001
	0.95 (0.92, 0.97)	0.95 (0.93, 0.98)	0.95 (0.92, 0.98)
**Marital Status**	p=<0.001	p=0.001	p=<0.001
Single	1.88 (0.89, 3.98)	12.4 (3.00, 51.2)	1.15 (0.50, 2.65)
Married	reference	reference	reference
Widowed	-	-	-
Divorced	2.12 (0.59, 7.69)	5.30 (0.82, 34.1)	2.42 (0.71, 8.28)
**Sex partners last 12m**	p<0.001	p<0.001	
1	reference	reference	reference
2-4	3.86 (1.51, 9.87)	5.55 (2.02, 15.2)	1.86 (0.78, 4.48)
5+	22.6 (5.56, 91.9)	13.4 (4.62, 38.6)	4.81 (1.66, 13.9)
**Partner type last intercourse**	p<0.001	p<0.001	
Stable	reference	reference	reference
Occasional	9.85 (4.14, 23.5)	9.81 (4.46, 21.6)	6.71 (2.84, 15.8)
Sex worker	25.1 (2.35, 270)	5.94 (0.61, 57.5)	11.5 (1.42, 93.4)

Multivariable model includes age, number of sex partners and type of partner, p value from Wald test.

Abbreviations: CI, confidence interval; OR odds ratio; CHF, Swiss Francs; yrs, years;

Table S9. Condom use, women reporting any same-sex partner. Associations with sociodemographic factors, sexual behaviours and substance use from univariable logistic regression, 2007, 2012, 2017 and from multivariable logistic regression 2007 and multivariable logistic regression 2012, 2017.

**Condom Use**	**2007**			**2012**		**2017**		
	**Prevalence (95 CI%)**	**Unadjusted OR (95% CI)**	**Adjusted OR (95% CI)** [a]	**Prevalence (95 CI%)**	**Unadjusted OR (95% CI)**	**Prevalence (95 CI%)**	**Unadjusted OR (95% CI**	**Adjusted OR (95% CI)** [a]
	22 (15-29)							
**Age, years**		p=0.025	p=0.12		p=0.009		p<0.001	p=0.011
Median (IQR)[b]	31 (24-43)	0.96 (0.92, 0.99)	0.97 (0.92, 1.01)	30 (22-41)	0.96 (0.93, 0.99	26 (23-31)	0.92 (0.88, 0.96)	0.96 (0.94, 0.98)
16-24	51 (22-79)	..	..	49 (33-66)	..	39 (21-60)	..	..
25-34	29 (17-45)	..	..	28 (17-42)	..	35 (22-51)	..	..
35-44	13 (7-22)	..	..	22 (12-35)	..	18 (7-40)	..	..
45-54	11 (5-21)	..	..	18 (9-32)	..	5 (1-16)	..	..
55-64	26 (9-57)	..	..	19 (3-51)	..	0 (0-0)	..	..
65-74	17 (0-91)	..	..	6 (0-69)	..	11 (0-0)	..	..
**Highest educational level**		p=0.7			p=0.6		p=0.5	
No school/primary	15 (9-39)	reference	..	37 (16-62)	reference	38 (10-73)	reference	..
Secondary	24 (15-34)	1.83 (0.26, 13.0)	..	25 (17-34)	0.57 (0.21, 1.58)	27 (17-39)	0.61 (0.16, 2.30)	..
Tertiary	20 (10-30)	1.42 (0.19, 10.4)	..	27 (18-38)	0.64 (0.22, 1.81)	21 (11-33)	0.44 (0.11, 1.69)	..
**Income, SFr**		p=0.8			p=0.025		p=0.9	
No Income	20 (0-43)	0.96 (0.20, 4.70)	..	10 (1-30)	0.28 (0.07, 1.10)	35 (3-83)	1.58 (0.33, 7.64)	..
<4500	21 (11-30)	reference	..	29 (21-37)	reference	26 (17-36)	reference	..
4500-6000	23 (9-37)	1.14 (0.43, 3.06)	..	32 (17-52)	0.57 (0.21, 1.58)	23 (7-46)	0.61 (0.16, 2.30)	..
>6000	32 (6-57)	1.79 (0.49, 6.52)	..	8 (2-21)	0.64 (0.22, 1.81)	21 (6-47)	0.44 (0.11, 1.69)	..
**Marital Status**		p<0.001			p<0.001		p<0.001	
Single	33 (22-44)	4.62 (1.43, 14.9)	..	39 (30-49)	4.36 (2.05, 9.27)	40 (17-69)	0.55 (0.17, 1.74)	..
Married	10 (0-19)	reference	..	13 (7-22)	reference	0 (0-0)	reference	..
Widowed	(0-0)	-	..	16 (0-46)	1.29 (0.12, 13.3)	11 (2-47)	-	..
Divorced	15 (5-24)	1.59 (0.42, 6.04)	..	15 (6-35)	1.39 (0.39, 5.02)	40 (17-69)	0.18 (0.03, 1.06)	..
**Age first intercourse, yrs**		p=0.8			p=0.043		p=0.11	
Median (IQR)[b]	17 (15-18)	1.02 (0.89, 1.16)	..	16 (15-17)	0.86 (0.74, 0.99)	16 (14-18)	0.89 (0.77, 1.03)	..
**Sex partners last 12m**		p<0.001	p=0.005		p<0.001		p<0.001	p<0.001
1	12 (06-19)	reference	reference	20 (14-27)	reference	11 (5-20)	reference	reference
2-4	45 (24-66)	5.94 (2.07, 17.1)	4.70 (1.55, 14.3)	61 (43-78)	6.49 (2.83, 14.9)	51 (33-70)	8.25 (2.98, 22.8)	5.96 (3.12, 11.4)
5+	57 (17-90)	9.66 (2.26, 41.3)	7.41 (1.42, 38.6)	56 (17-89)	5.11 (1.26, 20.7)	55 (5-97)	9.35 (1.64, 53.3)	4.99 (1.58, 15.7)
**Sex frequency last 12m**	Not asked	Not asked			p=0.005		p=0.08	
1-2 per year	..	..	..	40 (16-69)	reference	29 (0-97)	reference	..
1 per month	..	..	..	40 (20-62)	1.03 (0.27, 3.93)	19 (5-41)	1.75 (0.20, 15.6)	..
2-3 per month	..	..	..	43 (26-61)	1.13 (0.38, 3.38)	30 (13-51)	1.82 (0.44, 7.57)	..
1 per week	..	..	..	19 (10-31)	0.35 (0.12, 1.03)	31 (15-51)	1.98 (0.49, 7.99)	..
2-3 per week	..	..	..	12 (5-21)	0.2 (0.07, 0.61)	17 (5-35)	0.87 (0.19, 3.97)	..
4+ per week	..	..	..	36 (17-57)	1.03 (0.27, 3.93)	18 (1-57)	1.75 (0.20, 15.6)	..
**Partner type last intercourse**	Not asked	Not asked			p<0.001		p<0.001	
Stable	..	..	..	20 (15-27)	reference	15 (9-23)	reference	..
Occasional	..	..	..	72 (56-85)	10.2 (4.49, 23.1)	68 (45-87)	12.5 (4.12, 38.0)	..
Sex worker	..	..	..	0 (0-0)	-	0 (0-0)	-	..
**HIV testing**		p=0.11			p=0.9		p=0.2	
Never tested	17 (11-25)	reference	..	28 (17-41)	reference	24 (12-39)	reference	..
Tested >12-m	28 (15-45)	1.91 (0.79, 4.63)	..	29 (20-38)	1.06 (0.50, 2.23)	20 (11-32)	0.82 (0.31, 2.20)	..
Tested in last 12m	37 (15-65)	2.89 (0.92, 9.08)	..	25 (13-40)	0.87 (0.34, 2.22)	39 (21-59)	2.03 (0.69, 5.95)	..
**Alcohol**		p=0.6			p=0.6		p=0.074	
No use	21 (02-61)	reference	..	24 (10-43)	reference	9 (2-23)	reference	..
Daily	14 (03-36)	0.59 (0.07, 5.10)	..	13 (1-44)	0.46 (0.06, 3.48)	26 (1-76)	3.3 (0.46, 23.8)	..
Weekly	26 (17-36)	1.29 (0.21, 7.91)	..	30 (22-39)	1.39 (0.53, 3.65)	32 (22-44)	4.54 (1.37, 15.0)	..
Monthly	19 (09-32)	0.85 (0.13, 5.71)	..	25 (14-39)	1.08 (0.36, 3.27)	19 (8-35)	2.25 (0.57, 8.87)	..
**Tobacco**		p=0.7			p=0.14		p=0.022	
Non-smoker	21 (13-31)	reference	..	21 (13-30)	reference	14 (7-24)	reference	..
Casual smoker	31 (10-61)	1.69 (0.51, 5.61)	..	37 (22-53)	2.22 (0.95, 5.21)	39 (13-70)	3.79 (1.07, 13.5)	..
Daily smoker	22 (12-34)	1.02 (0.44, 2.37)	..	32 (21-43)	1.78 (0.85, 3.72)	35 (22-49)	3.21 (1.29, 7.95)	..
**Cannabis**[c]		p=0.7			p=0.069		p=0.038	

No use	17 (10-27)	reference	..	23 (15-34)	reference	16 (8-28)	reference	..
>12m ago	24 (14-36)	1.49 (0.62, 3.57)	..	23 (15-34)	1 (0.47, 2.13)	26 (15-39)	1.82 (0.68, 4.88)	..
Within last 12m	30 (14-52)	2.09 (0.72, 6.11)	..	41 (27-57)	2.3 (1.04, 5.06)	45 (24-67)	4.27 (1.39, 13.1)	..
**Other illicit drugs[c,d]**		p=0.09			p=0.2		p=0.6	
No use	19 (12-26)	reference	..	25 (18-32)	reference	42 (16-32)	reference	..
Any used	34 (16-51)	2.17 (0.88, 5.37)	..	35 (20-49)	1.61 (0.78, 3.34)	29 (12-46)	0.48, 3.30)	..

a. Multivariable models separate for 2007 and 2012, 2017 because denominator for 2007 excluded women who had never used a condom. Multivariable models include age, number of sex partners and type of partner; p value from Wald test;

b. Median age of the proportion who used a condom;

c. In 2007 questions about drugs were only asked to people between 15 to 69 years;

d. Combines use of ecstasy, cocaine, or heroin and combines categories of use more than 12 months ago and use within last 12 months.

Abbreviations: CI, confidence interval; IQR, interquartile range; m, months; OR, odds ratio; SFr, Swiss Francs; yrs, years; .., variable not included in multivariable model.

Table S10. Lifetime HIV testing, men and women with only opposite-sex partners. Denominators, unweighted and weighted, for each survey and variable

HIV testing	Men	Men	Men	Women	Women	Women
	2007	2012	2017	2007	2012	2017
**Totals, N unweighted, weighted[a]**	6236, 2237900	7063, 2313010	7975, 2702130	7582, 2289504	6763, 2049462	8330, 2529129
**Age, years**						
16-24	553, 296239	949, 290087	901, 300476	524, 248000	843, 255515	874, 258899
25-34	955, 384253	1040, 446058	1104, 525416	1183, 402818	1100, 419164	1278, 505920
35-44	1506, 505797	1402, 465779	1436, 525957	1753, 521670	1511, 471335	1604, 509388
45-54	1184, 441374	1629, 529873	1842, 566707	1344, 446311	1681, 504578	1921, 558324
55-64	1181, 365700	1179, 363623	1505, 461133	1532, 401480	1049, 307860	1592, 434936
65-74	857, 244536	864, 234265	1187, 322440	1246, 269225	579, 173550	1061, 290492
**Region**						
Lake Geneva	1165, 413941	1255, 421571	1412, 504218	1416, 429093	1223, 407290	1526, 479185
Midland	1651, 506532	1375, 521007	1563, 582248	2011, 519597	1332, 472081	1717, 593088
Northwest	657, 299390	984, 312365	842, 350702	806, 319638	944, 291493	889, 351691
Zurich	812, 398380	710, 397487	873, 485356	935, 386575	648, 344353	915, 443955
East	711, 334068	1067, 356147	1510, 385186	789, 304118	962, 300103	1477, 341979
Central	806, 202729	1175, 222817	1222, 273641	1032, 233854	1157, 217857	1240, 241036
Ticino	434, 82861	1255, 421571	1412, 504218	593, 96630	497, 98827	566, 107024
**Highest education level**						
No school/primary	613, 255693	808, 262368	941, 289892	1182, 347612	857, 275746	1019, 288781
Secondary	3443, 1220851	3436, 1132883	3756, 1256566	5039, 1523082	4163, 1283197	4726, 1400556
Tertiary	2179, 761150	2803, 930103	3264, 1151381	1358, 417617	1725, 567319	2566, 860448
Missing	1, 206	16, 4330	14,4291	3,1193	18, 5740	19, 8173
**Income, SFr**						
No income	149, 64349	244, 75987	342, 111526	889, 294891	888, 279067	963, 271987
<4500	2134, 789202	2253, 759570	2660, 910239	4367, 1346627	3897, 1221547	4896, 1466363
4500-6000	1520, 523117	1648, 549727	1881, 649750	862, 241467	699, 222686	1042, 356014
>6000	1769, 630922	2154, 697210	2503, 842885	407, 109029	443, 132703	732, 251417
Missing	664, 230310	764, 247191	589,187729	1057,297489	836, 276000	697, 212177
**Marital status**						
Single	1790, 702281	2230, 829308	2397, 991450	1776, 572834	1751, 585228	2247, 795789
Married	3600, 1323818	4208, 1263895	4859, 1430224	4065, 1353870	4226, 1262541	5024, 1414870
Widowed	149, 31389	80, 27635	70, 24647	684, 122382	126, 40937	206, 59874
Divorced	695, 179067	543, 208182	649, 255809	1054, 239916	658, 241865	853, 287426
Missing	2, 1347	2,664	0,0	3,502	2, 1431	0,0
**Sex partners last 12m**						
1	4883, 1793871	6086, 2005936	6437, 2140357	5811, 1908525	6331, 1924121	6876,1924121
2-4	666, 242075	712, 236900	660, 254133	404, 123338	384, 118102	488,118102
5+	200, 76497	221, 74953	197, 78844	34, 10340	29, 8387	29,8387
Missing	487, 106440	44,11896	681, 228796	1333, 110513	19, 6301	1053, 320588
**Condom use last intercourse**						
Not used	4946, 490230	5424, 1772851	6027, 1758067	6409, 350995	5567, 1688823	6710, 1997371
Used	1270, 654750	1630, 553994	1934, 552103	764, 726368	1188, 365366	1603, 526907
Missing	20, 6757	9, 2838	15, 4156	32, 9581	8, 2721	1209, 4850
**Alcohol**						
No use	504, 182855	557, 208127	802, 280558	1370, 404354	1082, 329781	1504, 461747
Daily	1329, 415464	1185, 354083	1161, 348790	747, 195648	526, 146025	516, 142503
Weekly	3301, 1, 235, 3	4002, 1310690	4589, 1571742	2905, 921169	3032, 926154	3924, 1215081
Monthly	1097, 401590	1318, 456511	1421, 500330	2558, 767830	2122, 654757	2385, 500330
Missing	5, 2653	1,273	2, 711	2, 504	1, 93	1,182
**Tobacco**						

Non-smoker	4122, 1465671	4675, 1512349	5437, 1791473	5614, 1703513	4994, 1512349	6243, 1896416
Casual smoker	599, 216270	694, 233922	768, 285872	227, 145214	463, 233922	596, 193855
Daily smoker	1515, 555960	1693, 582120	1770, 624782	622, 440333	1306, 582120	1490, 465636
Missing	0, 0	1, 1293	0,0	2, 444	0, 0	1,222
**Cannabis**[b]						
No use	4203, 1509223	4808, 1540934	5492, 1753661	5797, 1778908	5406, 1540934	6460, 1887252
Any use	1612, 614244	1985, 702051	2453, 936771	1184, 384976	1133, 702051	1857, 638014
Missing	421, 114433	270, 86968	30, 11698	588, 125620	224, 65444	13, 3863
**Other illicit drugs**[b,c]						
No use	5520, 2020619	6663, 2185257	7400, 2459983	6821, 2113861	6549, 2185257	8026, 2416588
Any use	313, 107355	393, 141195	562, 236133	173, 54604	212, 141195	300, 111683
Missing	403, 109927	7, 3231	13, 6013	588,121040	2, 371	4, 858

a. Overall denominators for condom use and HIV testing differ because eligibility criteria for answering each question differed;

b. In 2007 questions about drugs were only asked to people between 15 to 69 years;

c. Combines use of ecstasy, cocaine, or heroin

Abbreviations: m, months; SFr, Swiss Francs; yrs, years

Table S11. Lifetime HIV testing and associations from univariable and multivariable logistic regression, by sociodemographic and behavioural characteristics, in men reporting only opposite-sex partners 2007, 2012 and 2017

	Men 2007		Men 2012		Men 2017		
**HIV Testing**	**Prevalence (95% CI)**	**Unadjusted OR [a] (95% CI)**	**Prevalence (95% CI)**	**Unadjusted OR [a] (95% CI)**	**Prevalence (95% CI)**	**Unadjusted OR [a] (95% CI)**	**Adjusted OR (95% CI) [a]**
**Age, years**		p<0.001		p<0.001		p<0.001	p<0.001
Median (IQR)[b]	39 (31-49)	0.98 (0.98, 0.99)	42 (32-50)	0.99(0.98,0.99)	43 (31-47)	0.99(0.98,0.99)	0.99 (0.98,0.99)
16-24	20 (17-24)	..	29 (25-33)	..	40 (37-44)	..	..
25-34	50 (46-54)	..	52 (48-56)	..	47 (43-50)	..	..
35-44	42 (39-45)	..	55 (52-58)	..	57 (54-60)	..	..
45-54	33 (30-36)	..	44 (41-47)	..	53 (50-56)	..	..
55-64	21 (18-24)	..	33 (29-35)	..	37 (34-40)	..	..
65-74	12 (9-14)	..	21 (18-25)	..	24 (21-27)	..	..
**Region**		p<0.001		p<0.001		p<0.001	p<0.001
Lake Geneva	43 (40-47)	reference	54 (51-57)	reference	55 (53-58)	reference	reference
Midland	28 (25-31)	0.51 (0.42, 0.62)	37 (33-40)	0.50 (0.41, 0.60)	42 (39-45)	0.59 (0.50, 0.69)	0.54 (0.49, 0.62)
Northwest	30 (26-35)	0.58 (0.46, 0.73)	38 (34-41)	0.52 (0.42, 0.63)	42 (38-45)	0.58 (0.48, 0.70)	0.55 (0.48, 0.64)
Zurich	34 (31-38)	0.69 (0.56, 0.85)	48 (44-52)	0.79 (0.64, 0.98)	48 (45-52)	0.76 (0.63, 0.91)	0.66 (0.57, 0.76)
East	23 (19-27)	0.39 (0.30, 0.50)	35 (31-39)	0.46 (0.37, 0.58)	(39-36)	0.52 (0.44, 0.61)	0.44 (0.38, 0.51)
Central	31 (27-35)	0.59 (0.46, 0.75)	35 (31-38)	0.45 (0.37, 0.56)	39 (35-43)	0.51 (0.42, 0.62)	0.50 (0.43, 0.57)
Ticino	32 (27-37)	0.62 (0.47, 0.81)	47 (42-53)	0.78 (0.61, 0.99)	44 (39-49)	0.63 (0.51, 0.79)	0.78 (0.66, 0.91)
**Highest educational level**		p<0.001		p<0.001		p<0.001	p<0.001
No school/primary	21 (17-25)	reference	29 (25-33)	reference	32 (29-36)	reference	reference
Secondary	29 (27-31)	1.55 (1.20, 2.02)	41 (38-43)	1.69 (1.35, 2.11)	41 (39-43)	1.49 (1.25, 1.79)	1.69 (1.45,1.97)
Tertiary	40 (37-42)	2.46 (1.89, 3.21)	47 (45-49)	2.18 (1.74, 2.72)	52 (50-54)	2.34 (1.95, 2.80)	2.43 (2.06,2.86)
**Income, SFr**		p<0.001		p<0.001		p<0.001	p<0.001
No Income	20 (13-29)	0.65 (0.39, 1.07)	30 (22-38)	0.66 (0.45, 0.97)	45 (39-51)	1.25 (0.96, 1.64)	0.73 (0.60,0.93)
<4500	28 (26-31)	reference	39 (36-42)	reference	40 (37-42)	reference	reference
4500-6000	33 (30-36)	1.28 (1.07, 1.54)	43 (40-46)	1.17 (0.98, 1.39)	44 (42-47)	1.21 (1.05, 1.41)	1.25 (1.12,1.39)
>6000	37 (35-40)	1.52 (1.28, 1.79)	46 (43-49)	1.33 (1.13, 1.55)	52 (50-54)	1.66 (1.45, 1.89)	1.55 (1.38,1.73)
**Marital Status**		p<0.001		p<0.001		p<0.001	
Single	38 (35-41)	1.70 (1.47, 1.98)	46 (44-49)	1.5 (1.31, 1.72)	49 (46-51)	1.43 (1.27, 1.61)	1.18 (1.05,1.33)
Married	26 (25-28)	reference	37 (35-38)	reference	40 (38-41)	reference	reference
Widowed	19 (11-30)	0.65 (0.34, 1.22)	41 (27-55)	1.17 (0.66, 2.08)	43 (29-58)	1.17 (0.65, 2.10)	1.50 (0.96,2.34)
Divorced	51 (46-56)	2.91 (2.33, 3.64)	56 (50-61)	2.27 (1.76, 2.92)	61 (57-66)	2.41 (1.97, 2.94)	2.46(2.10,2.88)
**Age first intercourse, yrs**		p<0.001		p<0.001		p<0.001	p<0.001
Median age (IQR)[b]	18 (17-19)	0.90 (0.88, 0.93)	18 (17-19)	0.91 (0.89, 0.93)	17 (16-18)	0.97 (0.96, 0.98)	0.95 (0.94,0.97)
**Sex partners last 12m**		p<0.001		p<0.001		p<0.001	p=0.014
1	30 (29-32)	reference	41 (39-42)	reference	45 (43-46)	reference	reference
2-4	42 (37-47)	1.64 (1.32, 2.05)	46 (41-51)	1.24 (1.00, 1.55)	54 (49-59)	1.46 (1.20, 1.77)	1.16 (1.00,1.35)
5+	48 (39-58)	2.13 (1.45, 3.13)	54 (45-62)	1.67 (1.16, 2.41)	57 (48-65)	1.63 (1.16, 2.30)	1.37 (1.06,1.75)
**Condom use last intercourse**		p=0.6		p=0.8		p=0.3	p<0.001

Not used	32 (30-36)	reference	41 (38-45)	reference	46 (44-49)	reference	reference
Used	33 (30-36)	1.05 (0.89, 1.24)	42 (40-44)	0.98 (0.84, 1.14)	45 (43-46)	1.07 (0.94, 1.21)	0.82 (0.73,0.92)
**Alcohol**		p=0.13		p=0.8		p<0.001	p=0.07
No use	29 (24-35)	reference	41 (36-47)	reference	41 (37-45)	reference	reference
Daily	30 (27-33)	1.01 (0.75, 1.35)	40 (37-44)	0.96 (0.73, 1.25)	38 (35-41)	0.88 (0.71, 1.10)	0.80 (0.67,0.96)
Weekly	33 (32-35)	1.21 (0.92, 1.57)	42 (40-44)	1.02 (0.80, 1.29)	47 (45-49)	1.27 (1.06, 1.53)	0.82 (0.70,0.94)
Monthly	31 (27-34)	1.07 (0.79, 1.43)	43 (39-47)	1.06 (0.81, 1.39)	46 (43-49)	1.24 (1.01, 1.53)	0.94 (0.80,1.12)
**Tobacco**		p<0.001		p<0.001		p=0.03	p=0.5
Non-smoker	28 (27-30)	reference	39 (37-40)	reference	43 (42-45)	reference	reference
Casual smoker	38 (33-42)	1.52 (1.22, 1.89)	45 (40-50)	1.28 (1.03, 1.59)	50 (46-54)	1.29 (1.08, 1.56)	0.99 (0.86,1.14)
Daily smoker	39 (36-42)	1.61 (1.38, 1.88)	47 (43-50)	1.38 (1.19, 1.59)	48 (45-50)	1.18 (1.04, 1.34)	1.07 (0.97,1.18)
**Cannabis**[c]		p<0.001		p<0.001		p<0.001	p<0.001
No use	27 (25-29)	reference	35 (33-37)	reference	38 (37-40)	reference	reference
Any use	48 (44-51)	2.46 (2.12, 2.86)	56 (53-59)	2.36 (2.06, 2.71)	58 (55-60)	2.22 (1.98, 2.50)	1.62 (1.46,1.78)
**Other illicit drugs**[c,d]		p<0.001		p<0.001		p<0.001	p<0.001
No use	36 (35-37)	reference	40 (39-42)	reference	43 (42-44)	reference	reference
Any use	79 (71-85)	4.62 (3.41, 6.27)	65 (59-71)	2.78 (2.09, 3.69)	66 (61-71)	2.60 (2.10, 3.23)	1.72 (1.43,2.06)

a. p value from Wald test;

b. Median age of the proportion tested for HIV;

c. In 2007 questions about drugs were only asked to people between 15 to 69 years;

d. Combines use of ecstasy, cocaine, or heroin and combines categories of use more than 12 months ago and use within last 12 months.

Abbreviations: CI, confidence interval; m, months; OR, odds ratio; SFr, Swiss Francs; yrs, years;

Table S12. Prevalence of lifetime HIV testing and associations from univariable and multivariable logistic regression, by sociodemographic and behavioural characteristics, in women reporting only opposite-sex partners 2007, 2012 and 2017

	Women 2007		Women 2012		Women 2017		Adjusted OR (95% CI) [a]
**HIV Testing**	**Prevalence (95% CI)**	**Unadjusted OR [a] (95% CI)**	**Prevalence (95% CI)**	**Unadjusted OR [a] (95% CI)**	**Prevalence (95% CI)**	**Unadjusted OR[a] (95% CI)**	
**Age, years**		p<0.001		p<0.001		p<0.001	p<0.001
Median (IQR)[b]	38 (30-45)	0.96 (0.95, 0.96)	40 (31-47)	0.97 (0.97, 0.97)	43 (32-53)	0.98 (0.97, 0.98)	0.97 (0.96,0.97)
16-24	33 (28-38)	..	34 (30-38)	..	35 (32-39)	..	
25-34	56 (52-59)	..	63 (60-67)	..	62 (59-65)	..	
35-44	55 (52-58)	..	63 (60-66)	..	70 (68-73)	..	
45-54	32 (29-35)	..	49 (46-52)	..	58 (55-60)	..	
55-64	15 (12-17)	..	22 (19-25)	..	38 (35-41)	..	
65-74	6 (5-8)	..	11 (08-15)	..	22 (19-25)	..	
**Region**		p<0.001		p<0.001		p<0.001	p<0.001
Lake Geneva	47 (43-50)	reference	59 (56-62)	reference	64 (62-67)	reference	reference
Midland	36 (34-39)	0.65 (0.55,0.78)	44 (41-47)	0.55 (0.46, 0.66)	49 (47-52)	0.54 (0.46, 0.63)	0.58 (0.51, 0.65)
Northwest	31 (27-35)	0.51 (0.42,0.64)	42 (38-45)	0.5 (0.41, 0.61)	51 (47-54)	0.58 (0.48, 0.69)	0.51 (0.44, 0.58)
Zurich	38 (35-42)	0.71 (0.58,0.86)	48 (43-52)	0.63 (0.51, 0.78)	54 (51-58)	0.67 (0.56, 0.80)	0.56 (0.49, 0.64)
East	26 (22-30)	0.4 (0.32, 0.51)	41 (36-45)	0.47 (0.38, 0.59)	42 (39-45)	0.41 (0.34, 0.48)	0.42 (0.36, 0.48)
Central	28 (25-32)	0.45 (0.37,0.56)	39 (35-42)	0.44 (0.36, 0.53)	40 (36-44)	0.37 (0.30, 0.45)	0.42 (0.36, 0.48)
Ticino	33 (29-38)	0.58 (0.46,0.73)	47 (42-52)	0.63 (0.49, 0.79)	51 (46-55)	0.58 (0.47, 0.71)	0.62 (0.53, 0.73)
**Highest educational level**		p<0.001		p<0.001		p<0.001	p<0.001
No school/primary	19 (17-23)	reference	27 (24-31)	reference	35 (32-39)	reference	reference
Secondary	35 (33-36)	2.18 (1.78,2.67)	44 (42-46)	2.09 (1.69, 2.58)	47 (45-49)	1.63 (1.37, 1.93)	2.08 (1.81,2.38)
Tertiary	52 (49-56)	4.52 (3.59,5.70)	60 (58-63)	4.05 (3.21, 5.11)	64 (62-66)	3.31 (2.76, 3.96)	3.35 (2.87,3.92)
**Income, SFr**		p<0.001		p<0.001		p<0.001	p<0.001
No Income	31 (28-35)	0.87 (0.72,1.04)	43 (39-47)	0.91 (0.76, 1.09)	43 (39-47)	0.96 (0.81, 1.13)	0.91 (0.81,1.02)
<4500	34 (33-36)	reference	45 (43-47)	reference	45 (43-47)	reference	reference
4500-6000	49 (45-53)	1.82 (1.51,2.19)	57 (53-62)	1.65 (1.34, 2.03)	57 (53-62)	1.88 (1.60, 2.21)	1.42 (1.25,1.62)
>6000	56 (49-62)	2.39 (1.84,3.10)	61 (55-66)	1.89 (1.47, 2.43)	61 (55-66)	2.34 (1.93, 2.85)	1.64 (1.39,1.94)
**Marital Status**		p<0.001		p<0.001		p<0.001	p<0.001
Single	45 (42-48)	1.82 (1.58,2.11)	51 (48-54)	1.5 (1.31, 1.73)	58 (55-60)	1.65 (1.46, 1.86)	0.68 (0.60,0.76)
Married	31 (29-33)	reference	41 (39-43)	reference	45 (44-47)	reference	reference
Widowed	16 (12-21)	0.43 (0.31,0.60)	35 (24-46)	0.76 (0.46, 1.24)	33 (26-40)	0.58 (0.41, 0.81)	1.13 (0.83,1.55)
Divorced	47 (43-51)	1.98 (1.66,2.38)	64 (59-69)	2.51 (1.99, 3.15)	66 (62-70)	2.33 (1.96, 2.78)	2.33 (2.01,2.69)
**Age first intercourse, yrs**		p<0.001		p<0.001		p<0.001	p<0.001
Median age (IQR)[b]	18 (17-19)	0.88 (0.86,0.90)	17 (16-19)	0.88 (0.85, 0.90)	17 (16-19)	0.95 (0.93, 0.98)	0.96 (0.94,0.98)
**Sex partners last 12m**		p<0.001		p<0.001		p<0.001	p=0.8
1	37 (35-38)	reference	46 (44-47)	reference	52 (50-53)	reference	reference
2-4	50 (43-56)	1.69 (1.31,2.19)	57 (50-63)	1.56 (1.20, 2.02)	69 (63-74)	2.06 (1.60, 2.67)	1.06 (0.89,1.27)
5+	40 (21-62)	1.18 (0.51,2.72)	64 (38-85)	2.12 (0.81, 5.54)	74 (51-91)	2.71 (1.01, 7.30)	0.91 (0.51,1.63)
**Condom use last intercourse**		p=0.3		p= 0.8		p= 0.051	
Not used	34 (33-36)	reference	47 (43-50)	reference	54 (51-57)	reference	reference
Used	42 (39-46)	1. 41 (1.20,1.65)	46 (45-48)	1.02 (0.87, 1.20)	51 (49-52)	1.14 (1.00, 1.29)	0.79 (0.71,0.89)
**Alcohol**		p<0.001		p<0.001		p<0.001	p=0.03
No use	66 (63-69)	reference	47 (43-50)	reference	47 (44-50)	reference	reference
Daily	76 (72-80)	0.62 (0.48,0.80)	40 (34-45)	0.75 (0.58, 0.98)	39 (34-44)	0.72 (0.57, 0.92)	0.76 (0.63,0.92)
Weekly	61 (58-63)	1.27 (1.07,1.50)	48 (46-50)	1.05 (0.88, 1.25)	55 (53-57)	1.41 (1.23, 1.63)	0.91 (0.81,1.03)
Monthly	66 (63-68)	1.03 (0.86,1.22)	45 (43-48)	0.95 (0.79, 1.14)	50 (48-53)	1.16 (1.00, 1.36)	0.92 (0.82,1.04)
**Tobacco**		p<0.001		p<0.001		p<0.001	p=0.01
Non-smoker	33 (32-35)	reference	44 (42-45)	reference	49 (47-50)	reference	reference
Casual smoker	39 (34-45)	1.30 (1.01, 1.66)	56 (50-61)	1.65 (1.30, 2.08)	58 (53-62)	1.42 (1.17, 1.73)	0.95 (0.80,1.11)
Daily smoker	42 (39-46)	1.48 (1.27,1.71)	53 (50-56)	1.46 (1.25, 1.71)	59 (56-62)	1.49 (1.31, 1.71)	1.16 (1.04,1.29)
**Cannabis[c]**		p<0.001		p<0.001		p<0.001	p<0.001
No use	32 (30-33)	reference	41 (39-43)	reference	45 (44-46)	reference	reference
Any use	61 (58-65)	3.34 (2.84,3.93)	67 (63-70)	2.93 (2.47, 3.47)	70 (68-73)	5.33 (3.48, 8.14)	1.84 (1.64,2.06)
**Other illicit drugs[c, d]**		p<0.001		p<0.001		p<0.001	p<0.001
No use	36 (35-37)	reference	45 (44-47)	reference	50 (49-51)		reference
Any use	79 (71-85)	6.55 (4.33,9.91)	81 (74-87)	2.87 (2.52, 3.27)	83 (78-88)	5.02 (3.54, 7.14)	2.98 (2.20,4.02)

e. p value from Wald test;

f. Median age of the proportion tested for HIV;

g. In 2007 questions about drugs were only asked to people between 15 to 69 years;

h. Combines use of ecstasy, cocaine, or heroin and combines categories of use more than 12 months ago and use within last 12 months.

Abbreviations: CI, confidence interval; m, months; OR, odds ratio; SFr, Swiss Francs; yrs, years;

Table S13. HIV testing in the last 12 months, men and women reporting only opposite-sex partners and men reporting any same-sex partner 2012, 2017.

**HIV testing in the last 12 months**	**Men reporting only opposite-sex partners**		**Women reporting only opposite-sex partners**		**Men reporting any same-sex partner**	
	**2012**	**2017**	**2012**	**2017**	**2012**	**2017**
	**Prevalence % (95% CI)**	**Prevalence % (95% CI)**	**Prevalence % (95% CI)**	**Prevalence % (95% CI)**	**Prevalence % (95% CI)**	**Prevalence % (95% CI)**
Total	7 (6-8)	7 (7-8)	9 (8-10)	8 (7-9)	30 (14-38)	39 (30-47)
**Age, years**						
16-24	14 (11-18)	18 (15-21)	16 (13-19)	16 (13-19)	35 (11-70)	35 (17-59)
25-34	10 (8-13)	10 (8-12)	18 (15-20)	16 (13-18)	41 (23-62)	60 (39-79)
35-44	7 (6-9)	8 (6-10)	10 (8-12)	9 (7-11)	32 (19-48)	35 (19-55)
45-54	6 (5-8)	5 (4-6)	4 (3-5)	4 (3-5)	27 (14-45)	44 (26-63)
55-64	3 (2-4)	4 (3-5)	2 (1-4)	3 (2-4)	26 (11-50)	19 (9-37)
65-74	4 (2-5)	2 (1-3)	1 (0-3)	1 (1-2)	12 (2-49)	-
**Number of partners**						
1	5 (5-6)	5 (5-6)	8 (7-8)	7 (6-8)	20 (12-30)	31 (21-44)
2-4	17 (13-21)	18 (15-22)	28 (23-34)	31 (26-37)	42 (22-63)	48 (29-67)
5+	30 (21-40)	28 (21-36)	41 (20-65)	33 (16-56)	61 (37-82)	50 (31-69

Table S14. Lifetime HIV testing, men and women with any same-sex partners. Denominators, unweighted and weighted, and number of missing values for each survey and variable

HIV Testing	Men	Men	Men	Women	Women	Women
	2007	2012	2017	2007	2012	2017
**Totals, N unweighted, weighted[a]**	187, 64684	237, 81464	178,74070	216, 67259	289, 92734	172, 69018
**Age, years**						
16-24	7, 3497	20, 5643	25, 8197	20, 10605	54, 15237	14084, 8197
25-34	43, 17370	33, 14174	32, 17291	48, 17530	79, 30848	25382, 17291
35-44	58, 19554	68, 27822	38, 18051	64, 15196	62, 19610	12090, 18051
45-54	36, 11628	59, 18085	36, 14631	56, 18019	61, 17425	12713, 14631
55-64	31, 9701	38, 11063	37, 13623	22, 4563	23, 6537	3117, 13623
65-74	12, 2935	19, 4678	10, 2277	6, 1344	10, 3077	1631, 2277
**Region**						
Lake Geneva	29, 10,828	43, 14665	44, 18486	53, 13946	61, 19001	43, 16336
Midland	52, 17386	35, 15606	17, 8466	45, 11739	52, 17968	38, 18342
Northwest	17, 6329	43, 10864	19, 10322	34, 12394	56, 17660	16, 6521
Zurich	45, 18480	49, 24087	37, 19055	44, 17850	40, 21183	26, 14769
East	18, 5623	27, 9230	27, 10121	17, 5344	29, 7467	20, 6475
Central	14, 3722	34, 5939	26, 5903	19, 5549	36, 6356	20, 4442
Ticino	12, 2317	6, 1073	8, 1718	4, 437	15, 3100	9, 2133
**Highest educational level**						
No school/ primary	8, 3259	19, 4915	15, 4467	10, 2791	27, 8459	17, 5036
Secondary	98, 30528	106, 37877	80, 31484	131, 43428	152, 51662	83, 33636
Tertiary	81, 30897	112, 38672	83, 38119	75, 21040	110, 32612	72, 30346
Missing	0, 0	0, 0	0, 0	0, 0	0, 0	0, 0
**Income, SFr**						
No Income	5, 2205	9, 2488	12, 3984	14, 5575	19, 6716	8, 2581
<4500	66, 24662	64, 20121	62, 26067	121, 38545	178, 57005	106, 44156
4500-6000	51, 16664	49, 16453	42, 17768	42, 12900	37, 11380	31, 10373
>6000	51, 17320	732, 31517	50, 20141	24, 5869	29, 9141	22, 9965
Missing	14, 3833	32, 10885	12, 6110	15, 4370	26, 8492	5, 1943
**Marital status**						
Single	119, 41500	118, 45409	120, 52194	117, 35396	147, 48911	111, 47453
Married	41, 14123	96, 26288	18, 5042	47, 18229	99, 27702	22, 7883
Widowed	0, 0	1, 178	0, 0	4, 839	5, 1679	2, 428
Divorced	26, 7948	22, 9589	13, 5267	48, 12795	37, 13873	14, 5518
Missing	1, 1113	0, 0	27, 11566	0, 0	1, 569	23, 7735
**Sex partners last 12m**						
1	78, 28188	158,51901	86,34681	136, 45498	3367, 75583	106, 44848
2-4	62, 21451	44,17482	40,19451	36, 10840	253, 13385	41, 16743
5+	26, 10147	30,10335	37,14688	12, 3257	23, 3767	7, 3005
Missing	8, 4889	5, 1747	15, 5250	32, 7664	0, 0	18, 4422
**Condom use last intercourse**						
Used	80, 26642	150,53233	91,34347	51, 14040	213, 66891	129, 51731
Not used	107, 38042	86,27692	87,39903	165, 53219	76, 25843	43, 17287
Missing	0,0	1, 539	0, 0	0,0	0, 0	0, 0
**Alcohol**						
No use	17, 4041	28, 8021	12, 4354	20, 7383	32, 10546	225, 72098
Daily	37, 14060	36, 12419	28, 9542	23, 6067	24, 7291	64, 20636
Weekly	96, 31944	132, 46296	99, 44739	114, 35551	155, 48066	225, 72098
Monthly	37, 14638	41, 14729	39, 15436	58, 18006	78, 26831	64, 20636
Missing	0, 0	0, 0	0, 0	1,	0, 0	0, 0
**Tobacco**						
Non-smoker	102, 37373	137, 46754	103, 43499	102,37373	139, 47724	84, 34367

Casual smoker	25, 7274	30, 11727	18, 5952	25,7274	50, 16277	19, 7859
Daily smoker	60, 20037	70, 22983	57, 24620	60,20037	100, 28733	69, 26792
Missing	0, 0	0, 0	0, 0	0, 0	0, 0	0, 0
**Cannabis[b]**						
No use	90, 26874	125, 40575	91, 37982	91, 27166	86, 28336	74, 28862
Any use	90, 35547	110, 39979	85, 34967	89, 27750	203, 64398	96, 39422
Missing	7, 2264	2, 910	2, 1121	3, 1547	0, 0	2,734
**Other illicit drugs[b, c]**						
No use	138, 46,062	178, 57781	144, 60100	138,52314	225, 72,098	136, 53648
Any use	43, 16,637	54, 22233	34, 13970	43,14945	64, 20,636	36, 15370
Missing	6, 19860	5, 1543	0, 0	0, 0	0, 0	0, 0

a. Overall denominators for condom use and HIV testing differ because eligibility criteria for answering each question differed;

b. In 2007 questions about drugs were only asked to people between 15 to 69 years;

c. Combines use of ecstasy, cocaine, or heroin

Abbreviations: m, months; SFr, Swiss Francs; yrs, years

Table S15. Lifetime HIV testing and associations from univariable and multivariable logistic regression, testing, by sociodemographic and behavioural characteristics in men reporting any male sex partner, 2007, 2012, 2017

**HIV testing**		**2007**		**2012**		**2017**	
	**Prevalence (95% CI)**	**Unadjusted OR (95% CI)** [a]	**Prevalence (95% CI)**	**Unadjusted OR (95% CI)** [a]	**Prevalence (95% CI)**	**Unadjusted OR (95% CI) a**	**Adjusted OR (95% CI)** [a]
**Age**		p=0.072		p=0.3		p=0.9	p=0.5
Median (IQR)[b]	38 (30-48)	0.97 (0.94, 1.00)	42 (35-49)	0.98 (0.93, 1.02)	39 (32-52)	1.00 (0.97, 1.04)	0.99 (0.97, 1.02)
16-24	67 (00-100)	..	43 (14-77)	..	62 (38-82)	..	..
25-34	82 (64-92)	..	92 (76-97)	..	92 (69-98)	..	..
35-44	77 (61-88)	..	88 (72-95)	..	81 (62-92)	..	..
45-54	64 (38-83)	..	89 (78-95)	..	96 (85-99)	..	..
55-64	61 (36-81)	..	72 (53-86)	..	81 (61-92)	..	..
65-74	66 (30-90)	..	34 (14-63)	..	42 (08-87)	..	..
**Region**		p=0.2		p=0.049		p<0.001	
Lake Geneva	75 (54-89)	reference	83 (68-92)	reference	83 (68-92)	reference	..
Midland	68 (49-83)	0.71 (0.21, 2.37)	82 (65-92)	0.91 (0.28, 3.03)	82 (65-92)	0.66 (0.12, 3.78)	..
Northwest	84 (58-97)	1.81 (0.35, 9.48)	70 (44-87)	0.46 (0.12, 1.75)	70 (44-87)	0.47 (0.11, 1.91)	..
Zurich	77 (59-90)	1.16 (0.33, 4.11)	94 (83-98)	2.93 (0.75, 11.5)	94 (83-98)	1.82 (0.47, 7.07)	..
East	41 (15-70)	0.24 (0.05, 1.03)	60 (35-81)	0.3 (0.08, 1.07)	60 (35-81)	0.57 (0.15, 2.15)	..
Central	84 (46-99)	1.8 (0.23, 14.2)	70 (46-86)	0.46 (0.13, 1.62)	70 (46-86)	0.39 (0.11, 1.41)	..
Ticino	88 (59-99)	2.42 (0.37, 15.7)	67 (18-95)	0.40 (0.06, 2.83)	100	-	..
**Education level**		p=0.8		p=0.01		p=0.10	
No school/primary	73 (00-100)	reference	44 (19-73)	reference	83 (52-96)	reference	..
Secondary	70 (57-80)	0.86 (0.12, 6.06)	79 (67-87)	0.63 (0.16, 2.41)	75 (63-85)	0.63 (0.16, 2.41)	..
Tertiary	75 (61-85)	1.11 (0.16, 7.73)	87 (79-92)	1.84 (0.43, 7.89)	90 (80-95)	1.84 (0.43, 7.89)	..
**Income, SFr**		p=0.6		p=0.14		p=0.011	
No Income	54 (0-100)	0.69 (0.08, 6.37)	76 (0-100)	1.15 (0.22, 5.94)	66 (26-92)	0.66 (0.16, 2.64)	..
<4500	57 (42-72)	reference	74 (58-85)	reference	75 (60-86)	reference	..
4500-6000	40 (25-58)	0.79 (0.28, 2.25)	89 (79-95)	2.91 (1.02, 8.31)	92 (81-97)	3.85 (1.20, 12.4)	..
>6000	41 (26-58)	0.50 (0.18, 1.40)	87 (78-93)	2.48 (0.95, 6.47)	93 (82-97)	4.2 (1.29, 13.7)	..
**Marital Status**		p=0.001		p<0.001		p=0.8	
Single	80 (70-88)	4.51 (1.84, 11.1)	86 (78-92)	2.15 (0.96, 4.81)	81 (72-88)	0.95 (0.26, 3.42)	..
Married	47 (30-65)	reference	75 (64-83)	reference	82 (53-95)	reference	..
Widowed	-	-	100	-	-	-	..
Divorced	87 (65-96)	7.34 (1.88, 28.7)	68 (33-90)	0.83 (0.19, 3.61)	88 (57-98)	1.58 (0.26, 9.49)	..
**Age first sexual intercourse, years**		p= 0.038		p= 0.9		p= 0.5	
**Median (IQR)**	17 (15-19)	0.87 (0.76, 0.99)	17 (16-18)	0.99 (0.88, 1.11)	17 (16-18)	0.97 (0.89, 1.06)	..
**Sex partners 12m**		p<0.001		p=0.6		p=0.3	p=0.6
1	68 (53-79)	reference	77 (68-84)	reference	82 (70-89)	reference	Reference
2-4	73 (57-84)	1.26 (0.50, 3.18)	83 (66-92)	1.40 (0.53, 3.70)	91 (80-96)	2.29 (0.75, 6.96)	1.14 (0.61, 2.13)
5+	98 (91-100)	27.1 (4.99, 147)	89 (56-98)	2.43 (0.38, 15.7)	81 (59-93)	0.98 (0.29, 3.39)	1.65 (0.63, 4.30)
**Condom use last intercourse**		p=0.072		p=0.2		p=0.8	
Not used	69 (56-80)	reference	77 (68-84)	reference	84 (74-91)	reference	..
Used	83 (71-90)	2.60 (1.14, 5.95)	87 (75-93)	1.95 (0.78, 4.89)	83 (72-90)	0.87 (0.37, 2.08)	..
**Alcohol**		p=0.061		p=0.090		p=0.11	
No use	95 (72-99)	reference	63 (36-84)	reference	67 (29-91)	reference	..
Daily	60 (40-77)	0.09 (0.01, 0.56)	70 (44-87)	1.37 (0.31, 6.13)	72 (49-87)	1.27 (0.26, 6.18)	..
Weekly	76 (64-86)	0.19 (0.03, 1.11)	84 (77-90)	3.16 (1.04, 9.63)	84 (74-91)	2.66 (0.63, 11.2)	..
Monthly	70 (48-85)	0.13 (0.02, 0.88)	86 (71-94)	3.52 (0.93, 13.3)	92 (78-97)	5.64 (1.04, 30.7)	..
**Tobacco**		p=0.3		p=0.2		p=0.68	
Non-smoker	70 (58-80)	reference	80 (70-87)	reference	79 (68-87)	reference	..
Casual smoker	62 (36-82)	0.69 (0.23, 2.12)	69 (43-87)	0.55 (0.18, 1.72)	79 (49-94)	1.03 (0.29, 3.57)	..
Daily smoker	81 (64-91)	1.80 (0.67, 4.84)	87 (77-93)	1.71 (0.71, 4.09)	92 (83-97)	3.24 (1.16, 9.08)	..
**Cannabis**		p=0.023		p=0.010		p=0.9	
No use	59 (45-72)	reference	70 (59-79)	reference	84 (74-90)	reference	..
Any use	82 (71-90)	3.22 (1.36, 7.63)	91 (84-95)	3.86 (1.37, 10.8)	83 (70-91)	0.94 (0.39, 2.26)	..
**Other illicit drugs**[b]		p=0.019		p= 0.002		p=0.7	
No use	66 (55-75)	reference	75 (67-82)	reference	83 (75-88)	reference	..
Any use	90 (72-97)	4.86 (1.28, 18.4)	93 (85-97)	4.36 (1.67, 11.4)	87 (60-96)	1.35 (0.32, 5.70)	..

a. Multivariable model includes year, age and number of sex partners; p value from Wald test;

b. Median age of the proportion tested for HIV;

c. In 2007 questions about drugs were only asked to people between 15 to 69 years.

d. Combines use of ecstasy, cocaine, or heroin

Abbreviations: CI, confidence interval; IQR, interquartile range; m, months; OR, odds ratio; SFr, Swiss Francs; yrs, years; .., variable not included in multivariable model.

Table S16. Lifetime HIV testing, women reporting any same-sex partner, 2007, 2012, 2017. Associations with sociodemographic factors, sexual behaviours and substance use from univariable logistic regression

**HIV testing**	**2007**		**2012**		**2017**		
	**Prevalence (95% CI)**	**Unadjusted OR (95% CI)**	**Prevalence (95 CI%)**	**Unadjusted OR (95% CI)**	**Prevalence (95 CI%)**	**Unadjusted OR (95% CI)**	**Adjusted OR (95% CI)** [a]
**Year**							p=0.5
2007							reference
2012							1.35 (0.82, 2.23)
2017							1.31 (0.74, 2.30)
**Age**		p=0.020		p=0.7		p=0.8	p=0.9
Median age (years) (IQR)	36 (26-45)	0.96 (0.93, 0.99)	34(29-45)	0.99 (0.97, 1.02)	33 (26-44)	1.01 (0.97, 1.04)	1 (0.98, 1.02)
16-24	70 (38-90)	-	48 (32-65)	-	59 (37-77)	-	-
25-34	70 (54-83)	-	82 (69-90)	-	67 (51-80)	-	-
35-44	71 (55-83)	-	79 (64-89)	-	88 (68-96)	-	-
45-54	51 (33-68)	-	68 (52-81)	-	75 (55-88)	-	-
55-64	25 (09-54)	-	63 (31-87)	-	45 (05-93)	-	-
**Region**				p=0.5		p=0.3	
Lake Geneva	60 (43-76)	reference	68 (52-80)	reference	71 (53-84)	reference	..
Midland	64 (45-80)	1.14 (0.41, 3.21)	77 (59-88)	1.58 (0.57, 4.41)	64 (46-80)	0.74 (0.26, 2.12)	..
Northwest	63 (40-82)	1.10 (0.35, 3.41)	57 (41-72)	0.64 (0.26, 1.58)	56 (28-81)	0.53 (0.14, 2.01)	..
Zurich	54 (35-72)	0.77 (0.28, 2.09)	71 (52-85)	1.18 (0.42, 3.31)	80 (55-93)	1.69 (0.43, 6.57)	..
East	73 (38-94)	1.73 (0.37, 8.12)	79 (52-93)	1.79 (0.46, 6.94)	62 (29-87)	0.68 (0.15, 2.99)	..
Central	67 (29-93)	1.31 (0.26, 6.57)	78 (59-89)	1.66 (0.57, 4.84)	81 (55-94)	1.74 (0.45, 6.77)	..
Ticino	43 (0-100)	0.48 (0.04, 5.85)	75 (41-93)	1.43 (0.32, 6.37)	37(10-76)	0.24 (0.05, 1.22)	..
**Education level**		p=0.2		p=0.5		=0.3	
No school/primary	30 (04-84)	reference	56 (31-78)	reference	68 (34-90)	reference	..
Secondary	65 (54-75)	4.28 (0.87, 21.0)	69 (59-78)	1.78 (0.64, 4.93)	67 (53-78)	0.92 (0.26, 3.28)	..
Tertiary	57 (43-70)	3.03 (0.59, 15.5)	76 (64-84)	2.46 (0.85, 7.07)	71 (57-81)	1.11 (0.30, 4.07)	..
**Income, SFr**		p=0.5		p=0.7		p=0.3	
No Income	80 (39-96)	2.38 (0.47, 12.1)	86 (48-98)	3.48 (0.68, 17.8)	60 (13-94)	0.9 (0.17, 4.83)	..
<4500	62 (50-73)	reference	64 (55-72)	reference	63 (51-73)	reference	..
4500-6000	56 (35-75)	0.78 (0.30, 2.04)	64 (43-80)	1.00 (0.41, 2.43)	67 (44-84)	1.19 (0.44, 3.22)	..
>6000	51 (26-76)	0.63 (0.20, 1.94)	91 (75-97)	5.69 (1.69, 19.2)	93 (75-98)	7.84 (1.88, 32.8)	..
**Marital Status**		p=0.7		p=0.2		p=0.9	
Single	63 (51-74)	1.05 (0.46, 2.41)	73 (63-81)	1.48 (0.73, 2.96)	71 (60-80)	2.57 (0.87, 7.54)	..
Married	62 (45-76)	reference	64 (51-76)	reference	49 (24-74)	reference	..
Widowed	29 (0-80)	0.26 (0.02, 3.21)	76 (35-100)	1.74 (0.17, 17.5)	45 (0-100)	0.86 (0.04, 16.6)	..
Divorced	56 (35-75)	0.78 (0.27, 2.26)	71 (51-86)	1.26 (0.43, 3.64)	63 (21-92)	1.81 (0.35, 9.35)	..
**Age first sexual intercourse, years**		p=0.4		p=0.014		p<0.001	
**Median (IQR)**[b]	17 (15-18)	0.95 (0.84, 1.06)	16 (15-18)	0.86 (0.76, 0.97)	16 (15-18)	0.76 (0.64, 0.89)	..
**Sex partners 12m**		p=0.3		p=0.3		p<0.001	p=0.026
**1**	61 (51-71)	reference	68 (60-75)	reference	66 (54-76)	reference	reference
**2-4**	70 (48-86)	1.47 (0.55, 3.95)	80 (63-91)	1.92 (0.77, 4.80)	79 (60-91)	2 (0.73, 5.52)	1.79 (1.01, 3.17)
**5+**	87 (31-99)	4.32 (0.50, 37.3)	83 (27-99)	2.37 (0.29, 19.5)	100	-	4.65 (1.02, 21.1)
**Condom use last intercourse**		p=0.4		p=0.9		p=0.8	..
Not used	59 (49-68)	reference	70 (62-77)	reference	68 (58-77)	reference	..
Used	70 (52-83)	1.64 (0.70, 3.84)	70 (56-81)	0.99 (0.49, 2.03)	70 (51-84)	1.12 (0.46, 2.72)	..
**Alcohol**		p >0.9		p=0.4		p<0.001	..
No use	66 (36-88)	reference	55 (32-76)	reference	95 (83-99)	reference	..
Daily	61 (33-83)	0.79 (0.16, 3.77)	64 (35-85)	1.44 (0.35, 5.89)	46 (11-87)	0.05 (0.01, 0.31)	..
Weekly	60 (48-71)	0.75 (0.22, 2.60)	72 (63-80)	2.13 (0.78, 5.80)	71 (60-81)	0.13 (0.03, 0.52)	..
Monthly	62 (44-77)	0.82 (0.22, 3.12)	74 (60-84)	2.30 (0.78, 6.81)	54 (36-71)	0.06 (0.01, 0.27)	..
**Tobacco**		p=0.4		p=0.6		p= 0.5	..
Non-smoker	60 (47-71)	reference	68 (58-77)	reference	67 (54-78)	reference	..
Casual smoker	48 (23-73)	0.61 (0.20, 1.86)	67 (49-81)	0.41, 2.21	80 (50-94)	1.96 (0.58, 6.63)	..
Daily smoker	66 (51-78)	1.27 (0.58, 2.76)	75 (63-85)	0.68, 2.97	68 (53-80)	1.04 (0.46, 2.35)	..
**Cannabis**[c]		p=0.11		p=0.001		p<0.001	..
No use	50 (37-63)	reference	53 (40-66)	reference	51 (37-64)	reference	..
Any use	70 (57-80)	2.31 (1.06, 5.06)	78 (69-84)	3.04 (1.54, 5.97)	82 (71-90)	4.46 (1.92, 10.4)	..
**Other illicit drugs**[d]		p=0.3		p=0.13		p=0.2	..
No use	59 (49-68)	reference	67 (59-74)	reference	65 (55-74)	reference	..

Any use	69 (50-83)	1.55 (0.65, 3.69)	81 (63-92)	2.09 (0.79, 5.53)	80 (58-93)	2.21 (0.71, 6.89)	..

e. Multivariable model includes age, and number of sex partners; p value from Wald test;

f. Median age of the proportion tested for HIV;

g. In 2007 questions about drugs were only asked to people between 15 to 69 years;

h. Combines use of ecstasy, cocaine, or heroin and combines categories of use more than 12 months ago and use within last 12 months.

Abbreviations: CI, confidence interval; IQR, interquartile range; m, months; OR, odds ratio; SFr, Swiss Francs; yrs, years; .., variable not included in multivariable model.
